# Integrative metabolomics of plasma and PBMCs identifies distinctive metabolic signatures in Behçet’s disease

**DOI:** 10.1186/s13075-022-02986-5

**Published:** 2023-01-07

**Authors:** Soo Jin Park, Mi Jin Park, Sun Park, Eun-So Lee, Do Yup Lee

**Affiliations:** 1 Department of Agricultural Biotechnology, Seoul National University, Seoul, Republic of Korea; 2 Department of Dermatology, Ajou University School of Medicine, 164 Worldcup-ro, Yeongtong-gu, Suwon, 16499 Republic of Korea; 3 Department of Microbiology, Ajou University School of Medicine, Suwon, 16499 Republic of Korea; 4 Center for Food and Bioconvergence, Research Institute for Agricultural and Life Sciences, College of Agriculture and Life Sciences, Seoul National University, 1 Gwanak-ro, Gwanak-gu, Seoul, 08826 Republic of Korea

**Keywords:** Metabolomics, Lipidomics, Behçet’s disease, Autoimmune disease

## Abstract

**Background:**

Behçet’s disease (BD) is a systemic inflammatory disease that involves various organs. The clinical manifestation-based diagnosis of BD is a time-consuming process, which makes it difficult to distinguish from patients with similar symptoms. Moreover, an authentic biomarker has not been developed for accurate diagnosis yet. Our current study investigated the unique metabolic signatures of BD and explored biomarkers for precise diagnosis based on an untargeted metabolomic approach.

**Methods:**

Integrative metabolomic and lipidomic profiling was performed on plasma samples of BD patients (*n* = 40), healthy controls (HCs, *n* = 18), and disease controls (DCs, *n* = 17) using GC-TOF MS and LC-Orbitrap MS. Additionally, the lipid profiles of 66 peripheral blood mononuclear cells (PBMCs) were analyzed from 29 BD patients, 18 HCs, and 19 DCs.

**Results:**

Plasma metabolic dysfunction in BD was determined in carbohydrate, hydroxy fatty acid, and polyunsaturated fatty acid metabolisms. A plasma biomarker panel with 13 compounds was constructed, which simultaneously distinguished BD from HC and DC (AUCs ranged from 0.810 to 0.966). Dysregulated PBMC metabolome was signatured by a significant elevation in lysophosphatidylcholines (LPCs) and ether-linked lysophosphatidylethanolamines (EtherLPEs). Ten PBMC-derived lipid composites showed good discrimination power (AUCs ranged from 0.900 to 0.973). Correlation analysis revealed a potential association between disease activity and the metabolites of plasma and PBMC, including sphingosine-1 phosphate and EtherLPE 18:2.

**Conclusions:**

We identified metabolic biomarkers from plasma PBMC, which selectively discriminated BD from healthy control and patients with similar symptoms (recurrent mouth ulcers with/without genital ulcers). The strong correlation was determined between the BD activity and the lipid molecules. These findings may lead to the development for diagnostic and prognostic biomarkers based on a better understanding of the BD pathomechanism.

**Supplementary Information:**

The online version contains supplementary material available at 10.1186/s13075-022-02986-5.

## Background

Behçet’s disease (BD) is a chronic multisystemic inflammatory disease. The disorder is characterized by recurrent oro-genital, ocular, and skin lesions and by the involvement of multiple joints, the gastrointestinal tract, the cardiovascular system, and the central nervous system [1, 2]. Abnormal immunity has been described as the cause of BD; however, the etiology of BD has not yet been fully elucidated. Dysregulation of the innate and adaptive immune systems is induced by various attributor, such as genetic, microbial, and autoimmune factors, in BD [3, 4]. Genetic predisposition (MHC class I locus HLA-B51) is considered to play a critical role in the development of the disease, but genetic components are not sufficient to explain the occurrence of BD [3]. Instead, environmental factors appear to play a crucial role. An infectious agent may be required to trigger an innate inflammatory response, and autoantigen-activated antigen-presenting cells may also sustain an adaptive response [5]. Self-reactive autoinflammation induces the secretion of proinflammatory cytokines (interleukin (IL)-1, IL-8, and tumor necrosis factor-alpha (TNF-α)), which results in T-cell activation and tissue damage in BD [6]. However, the mechanisms underlying BD remain unclear.

BD diagnosis is solely based on clinical signs and symptoms. Diagnostic criteria have been developed, but there are no specific laboratory tests, imaging, or biopsy findings [7, 8]. Some BD symptoms are common in various disorders, making correct diagnosis more difficult. For instance, recurrent oro-genital ulceration is regarded as an important clinical manifestation in BD diagnosis, but it is not specific or sensitive enough to differentially diagnose from diseases with similar symptoms (e.g., complex aphthosis and PFAPA syndrome). BD diagnosis needs a careful evaluation of medical history, which may take several years. Accordingly, it is necessary to investigate and develop BD-specific laboratory findings by comparably analyzing patients with recurrent mouth ulcers with/without genital ulcers.

Monitoring of BD activity is critical because unpredictable and repetitive exacerbations and remissions characterize BD progression. Although the erythrocyte sedimentation rate (ESR) and C-reactive protein (CRP) levels have been regarded as activity markers [9], no marker correlates substantially with disease status.

In this regard, clinical metabolomics is an ideal platform for elucidating the molecular mechanisms underlying the development of diseases and screening biomarkers for diagnosis and prognosis. A few BD biomarkers have been proposed based on a metabolomic approach, but specific biomarkers have not yet been developed [10–17].

Accordingly, we metabolically characterized the blood and blood and peripheral blood mononuclear cells (PBMCs) of BD patients based on untargeted metabolomic and lipidomic profiling. Unique metabolic features were identified and reconstructed for diagnostic biomarkers that discriminate BD patients simultaneously from healthy controls and patients with similar symptoms (recurrent mouth ulcers with/without genital ulcers). Furthermore, we determined metabolic alterations according to disease activity and identified activity-dependent metabolic features.

## Materials and methods

### Demographic characteristics of participants

We enrolled patients with BD, recurrent aphthous ulcer (RAU) only, or RAU with either recurrent genital ulcer or erythema nodosum as disease controls (DCs) and healthy controls (HCs) who presented for the first time or were being monitored at the outpatient clinic of the Department of Dermatology, Ajou University Hospital, between March 2015 and October 2016. BD was diagnosed using the criteria of the International Study Group or Behçet’s Syndrome Research Committee of Japan [18, 19]. A total of 40 BD patients and 17 DCs were enrolled for plasma sampling; 29 BDs and 19 DCs were enrolled for PBMC sampling. The baseline clinical characteristics are summarized in Table 1. All BD patients had more than three clinical manifestations at diagnosis, including oral ulcers, genital ulcers, skin manifestations, and ocular inflammation. Joint, gastrointestinal, neurological, and vascular symptoms were less common. Patients with BD, infectious or inflammatory diseases, and other systemic diseases, including liver or renal failure, diabetes mellitus, and malignancies, and those with insufficient medical records were excluded. The patients with active BD converted to the disease-inactive phase, which was defined as the period in which patients exhibited no mucocutaneous or systemic lesions and no other BD-related findings and had inflammatory marker levels following systemic treatment lower than those at the initial visit. The DC group consisted of 27 age-matched patients with only two mucocutaneous symptoms or recurrent aphthous ulcers. These patients were often misdiagnosed with BD. The HC group consisted of 18 healthy age-matched volunteers. Informed consent was obtained from all the participants prior to enrollment. This study was approved by the Institutional Review Board of Ajou University Hospital (IRB number: AJIRB-BMR-GEN-14-463).

**Table 1:** Clinical characteristics of the participants

Group	Plasma			PBMC		
	HC^a^	BD	DC	HC^a^	BD	DC
	(*N*=18)	(*N*=40)	(*N*=17)	(*N*=18)	(*N*=29)	(*N*=19)
Age (years), mean ± SD	45 ± 7.7	44 ± 8	46 ± 11.7	45 ± 7.7	47 ± 9.7	46 ± 12.5
Age at BD diagnosis (years), mean ± SD		34 ± 7.3	36 ± 13.6		37 ± 9.4	38 ± 12.8
Sex						
**Male,** ***N*** **(%)**	2 (11.1)	21 (52.5)	6 (35.3)	2 (11.1)	18 (62.1)	8 (42.1)
**Female,** ***N*** **(%)**	16 (88.9)	19 (47.5)	11 (64.7)	16 (88.9)	11 (37.9)	11 (57.9)
BMI	23 ± 3.2	24 ± 3.0	24 ± 2.7	23 ± 3.2	22 ± 3.3	24 ± 1.8
Disease characteristics						
**Oral aphthous ulcers,** ***N*** **(%)**	0 (0)	39 (97.5)	17 (100)	0 (0)	28 (96.6)	19 (100)
**Genital ulcers,** ***N*** **(%)**	0 (0)	29 (72.5)	7 (41.2)	0 (0)	18 (62.1)	3 (15.8)
**Skin lesions,** ***N*** **(%)**	0 (0)	37 (92.5)	12 (70.6)	0 (0)	27 (93.1)	14 (73.7)
**Uveitis,** ***N*** **(%)**	0 (0)	14 (35)	1 (5.9)	0 (0)	12 (41.4)	1 (5.3)
**Arthritis,** ***N*** **(%)**	0 (0)	6 (15)	1 (5.9)	0 (0)	2 (6.9)	1 (5.3)
**GI lesions,** ***N*** **(%)**	0 (0)	1 (2.5)	2 (11.8)	0 (0)	1 (3.4)	3 (15.8)
**CNS lesions,** ***N*** **(%)**	0 (0)	2 (5)	1 (5.9)	0 (0)	1 (3.4)	1 (5.3)
**Vascular involvement,** ***N*** **(%)**	0 (0)	8 (20)	0 (0)	0 (0)	6 (20.7)	0 (0)
**Dyslipidemia,** ***N*** **(%)**	5 (27.8)	3 (7.5)	1 (5.9)	5 (27.8)	0 (0)	1 (5.3)
**Positive ANA,** ***N*** **(%)**	0	32	17	0	8 (27.6)	19
Pathergy test	0 (0)	10 (31.3)	1 (5.9)	0 (0)	21	1 (5.3)
**Positive pathergy,** ***N*** **(%)**	0 (0)	3 (7.5)	1 (5.9)	0 (0)	9 (42.9)	1 (5.3)
HLA-B51 test	0	37	17	0	28	17
**Positive,** ***N*** **(%)**	0 (0)	19 (51.4)	8 (47.1)	0 (0)	15 (53.6)	7 (41.2)
**Negative,** ***N*** **(%)**	0 (0)	18 (45)	9 (52.9)	0 (0)	13 (44.8)	10 (52.6)
Previous treatment						
**Systemic steroids,** ***N*** **(%)**	0 (0)	9 (22.5)	1 (5.9)	0 (0)	4 (13.8)	0 (0)
**Colchicine,** ***N*** **(%)**	0 (0)	25 (62.5)	5 (29.4)	0 (0)	24 (82.8)	2 (10.5)
**Azathioprine,** ***N*** **(%)**	0 (0)	6 (15)	0 (0)	0 (0)	5 (17.2)	0 (0)
**Salazopyrine,** ***N*** **(%)**	0 (0)	7 (17.5)	0 (0)	0 (0)	9 (31)	0 (0)
**Pentoxyphylline,** ***N*** **(%)**	0 (0)	14 (35)	5 (29.4)	0 (0)	19 (65.5)	2 (10.5)
**Cilostazol,** ***N*** **(%)**	0 (0)	3 (7.5)	0 (0)	0 (0)	2 (6.9)	0 (0)
**Antibiotic,** ***N*** **(%)**	0 (0)	6 (15)	0 (0)	0 (0)	5 (17.2)	0 (0)
**Statin,** ***N*** **(%)**	0 (0)	2 (5)	0 (0)	0 (0)	0 (0)	0 (0)

*BD* Behçet’s disease, *GI* Gastrointestinal, *CNS* Central nervous system, *ANA* Antinuclear antibody, *HLA* Human leukocyte antigen, *N/A* Not applicable

^a^The HC is the same participant

### Plasma and PBMC collection

Blood samples were collected from the subjects in standard 10-mL heparin-treated vacutainer tubes (Vacutainer® tubes; Becton Dickinson, Stockholm, Sweden). Peripheral blood mononuclear cells (PBMCs) were isolated from each sample using Ficoll-Paque density gradient centrifugation (Ficoll-Paque™ plus; Stem Cell Technologies, Vancouver, BC, Canada).

### Metabolite extraction from plasma

Plasma samples were thawed on ice at 4°C, and an aliquot of 50 μL was used for the extraction with 650 μL of the extraction solvent (methanol: isopropanol: water, 3:3:2, v/v/v). The mixture was sonicated for 10 min and then centrifuged at 13,200 rpm for 5 min at 4°C. Each supernatant (700 μL) was transferred into a new 1.5-mL tube. Aliquots were concentrated and completely dried in a speed vacuum concentrator (SCANVAC, Korea). The dry extracts were stored at −80°C until derivatization and gas chromatography time-of-flight mass spectrometry (GC-TOF-MS) analysis.

### Lipid extraction from plasma and PBMC

Plasma samples (50 μL) were used for lipid extraction. The PBMC samples were freeze-dried and pulverized using a Mixer Mill MM400 (Retsch GmbH & Co., Germany). The lipid extraction procedure was conducted using a previously described method based on the Folch method with minor modifications [20, 21].

### Gas chromatography time-of-flight mass spectrometry for metabolite profiling

The dried extracts were derivatized with 5 μL of 40 mg/mL methoxyamine hydrochloride (Sigma-Aldrich, St. Louis, MO, USA) in pyridine (Thermo Fisher Scientific, USA) and incubated for methoxyamination (90 min at 200 rpm and 30°C). For the retention time index, 2 μL of a mixture of fatty acid methyl esters [FAMEs] was added and 45 μL of *N*-methyl-*N*-trimethylsilyltrifluoroacetamide (MSTFA + 1% TMCS; Thermo, USA) was combined for trimethylsilylation (1h at 200 rpm and 37°C). The FAME mixture was composed of C8, C9, C10, C12, C14, C16, C18, C20, C22, C24, C26, C28, and C30.

GC-MS analysis was performed as described previously [22]. Using an Agilent 7890B gas chromatograph system (Agilent Technologies) equipped with an RTX-5Sil MS column (Restek, Gellefonte, PA, USA), the derivatives (0.5 μL) were injected in splitless mode. The entire system was controlled using the ChromaTOF software 4.50 version (LECO, St. Joseph, MI, USA). Mass spectra were acquired in the 85–500 *m/z* at an acquisition rate of 20 spectra/s using a Leco Pegasus HT time-of-flight mass spectrometer.

### UPLC-Orbitrap MS analysis for lipid profiling

Details of the lipid profiling methods have been presented elsewhere [20, 23]. Briefly, a Q-Exactive Plus instrument (Thermo Fisher Scientific, MA, USA) equipped with an Ultimate-3000 UPLC system (Thermo Fisher Scientific, USA) was used for detection. Plasma and PBMCs were analyzed under negative mode and polarity switching, respectively. The raw data were processed using MS-DIAL for alignment and identification (MS1 tolerance, 0.005 Da; MS2, 0.05 Da, similarity score: 70%).

### Statistical analysis

Statistical analyses were conducted for all continuous variables. Missing value imputation and univariate statistics were performed using R (https://github.com/CHKim5/LMSstat). Chemical similarity enrichment analysis was performed using ChemRICH [24]. Partial least squares-discriminant analysis (PLS-DA), variable importance in projection (VIP), receiver operating characteristic (ROC) analysis, and shared and unique structure plots were performed using SIMCA 17 software (Umetrics AB, Umea, Sweden). PLS-DA was performed using a 7-fold cross-validation and permutation test (*N* = 999). For validation of the ROC analysis, 95% confidence interval using bootstrapping was performed using the biomarker analysis module implemented in Medcalc® (Medcalc® Software, Mariakerke, Belgium). Pathway enrichment analysis and pattern searching module were performed using MetaboAnalyst 4.0 (http://metaboanalyst.ca). Graphs were generated using Prism 8 (GraphPad Software, San Diego, CA, USA).

## Results

### The effects of potential confounding factors on blood and PBMC metabolome

Before statistical analysis, we estimated the potential confounding effects of sex, age, BMI, dyslipidemia, and, nine drugs on the blood and PBMC metabolome. Fisher’s exact test was used to identify confounding variables according to different groups (BD, HC, and DC). Sex, colchicine, salazopyrin, and pentoxifylline were significantly different (*p* < 0.05) between the groups in both plasma and PBMCs (Fig. S1A). The significant difference was not found in BMI between HC and BD patients (Mann-Whitney test, *p*-value = 0.253 in plasma and 0.683 in PBMC). Considering the potential effects on global metabolite profiles, potential confounders were further speculated based on principal component analysis (PCA). The score scatter plot of PCA showed no distinct clusters by the potential confounders (Fig. S1B). The results indicated that the confounders did not marginally affect or influence the blood and PBMC metabolome.

### Carbohydrate, fatty acid, and phosphatidylethanolamine metabolisms were significantly altered in the plasma of BD patients

First, we investigated the primary metabolite profiles of plasma samples. A total of 92 metabolites were identified using gas chromatography time-of-flight mass spectrometry (GC-TOF MS). The identified metabolites were classified as amino acids (28%), fatty acids (FAs) and conjugates (22%), sugars and sugar alcohols (14%), purines and pyrimidines (7%), organic acids (5%), and miscellaneous compounds.

**Fig. 1:**
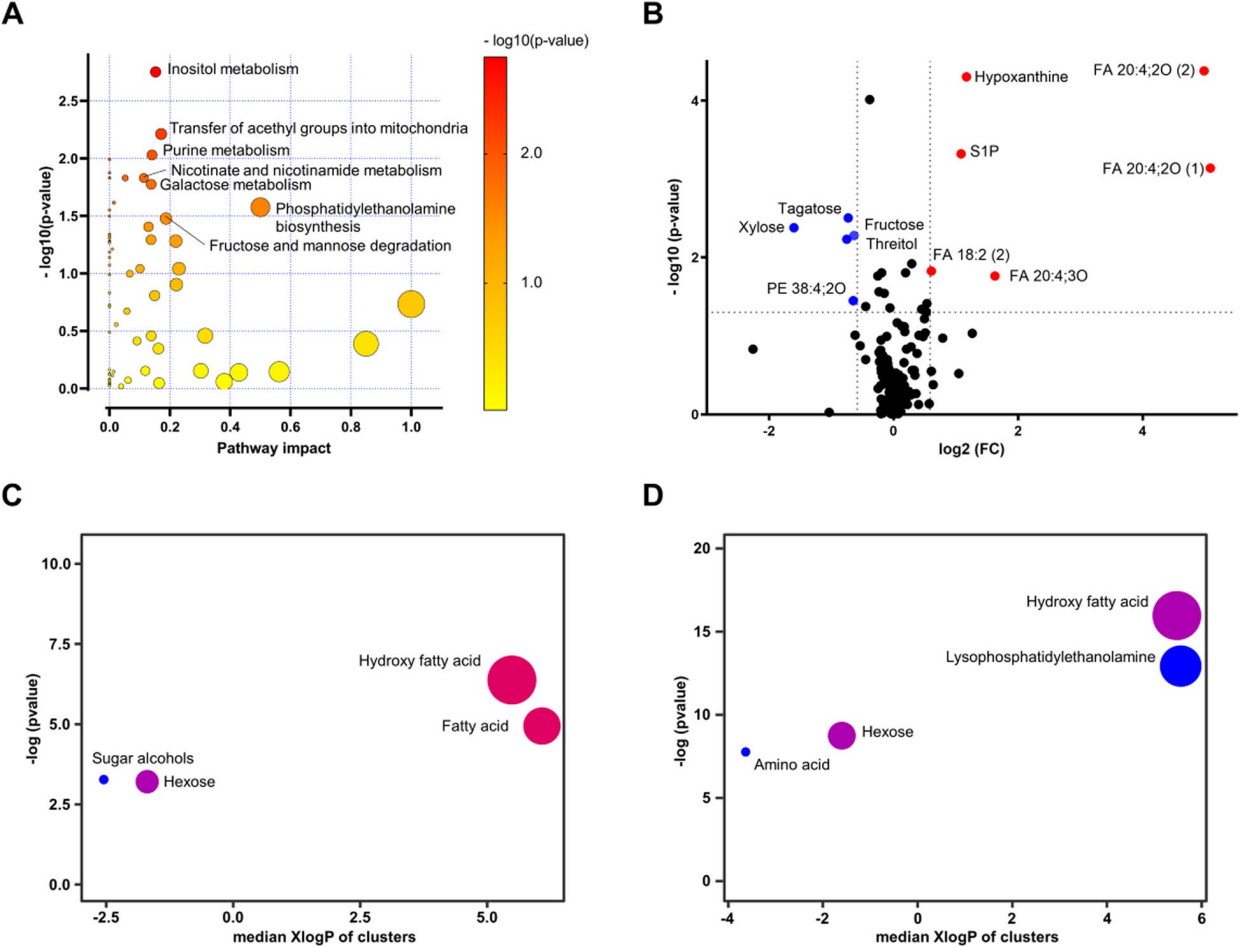
Distinctive blood plasma metabolomic profile in BD. **A** Pathway enrichment analysis showed pathway perturbation in BD compared to HC. The *X*-axis indicates pathway impact values (from pathway topology analysis), and the *Y*-axis indicates *p*-values (from pathway enrichment analysis). The node color is based on its *p*-value and the node radius is determined based on pathway impact. **B** Volcano plot for compounds altered in BD compared to that in HC. The *p*-value is calculated based on the Mann-Whitney test. Red and blue indicate a 1.5-fold increase and decrease, respectively, in BD. ChemRICH analysis of BD compared with HC (**C**) and DC (**D**). The node color scale represents the ratio of an increased (red) or decreased (blue) compound. The node size means the total number of metabolites in each cluster

To provide an overview of the dysregulation of metabolic pathways in BD, pathway enrichment analysis was performed based on the Small Molecule Pathway Database (SMPDB) (Fig. 1A). Carbohydrate metabolism, such as fructose-mannose degradation, inositol metabolism, and galactose metabolism, was significantly altered in BD compared to that in HCs. Significantly altered intermediates included glucose, fructose, tagatose, xylose, myo-inositol, and threitol (Mann-Whitney test, *p* < 0.05; Dunn test with Benjamini-Hochberg correction, *p* < 0.05) (Table S1). Dysregulation of carbohydrate metabolism coincided with a significant alteration in citric acid, whereas other TCA intermediates (fumaric acid and succinic acid) did not change (Table S1). Phosphatidylethanolamine (PE) biosynthesis showed the highest pathway impact among the significantly altered metabolisms (pyrophosphate, ethanolamine, *o*-phosphorylethanolamine, and serine).

Given the alteration of phospholipid biosynthesis in BD, we extended metabolic interrogation to the lipid profile. Untargeted lipid profiling was conducted using liquid chromatography Orbitrap mass spectrometry (LC-Orbitrap MS). Forty-six lipids were identified and quantified. Mann-Whitney and Dunn tests were performed on primary metabolites and lipid profiles to test statistical significance (Table S1). A total of 24 compounds, including hydroxy fatty acids (HFA), polyunsaturated fatty acids (PUFA), hexoses, and sugar alcohols, were significantly dysregulated in BD compared to that in HCs. FA 20:4;2O, oxidized arachidonic acid, showed the highest fold change (30 times higher in BD relative to HC) (Fig. 1B). Others included FA 20:4;3O, hypoxanthine, sphingosine 1-phosphate (S1P), and FA 18:2 (linolenic acid). The highest fold-decrease in BD was observed for xylose, threitol, tagatose, fructose, and oxidized phosphatidylethanolamine (OxPE) 38:4;2O (Fig. 1B).

### FA dysregulation was specific to BD against HC and DC

To identify metabolites with BD-specific alterations, we cross-validated the metabolites in the DC group. Ten compounds were exclusively altered only in BD patients, but not in DCs, compared to that in HC (Fig. S2). Unique metabolic changes (upregulation) were observed in the intermediates of PE metabolism (pyrophosphate, phenylalanine, PE 38:4;2O, and LPE O-18:2). Others were PUFAs, including FA 16:4, FA 18:2, and FA 28:7, which are exclusively dysregulated in BD.

Chemical similarity enrichment analysis (ChemRICH) was used to identify the enrichment of specific chemical classes based on structural similarity and metabolite enrichment significance. The HFA and FA clusters were the most significantly increased in the BD group compared to those in the HC group (Fig. 1C). A significant change in HFA was also detected in DC, but the FA levels did not change (Fig. 1D). FA 16:4 was significantly upregulated in BD compared to that in HC and DC (Table S1).

### The plasma metabolite panel discriminates BD from HC and DC

**Fig. 2:**
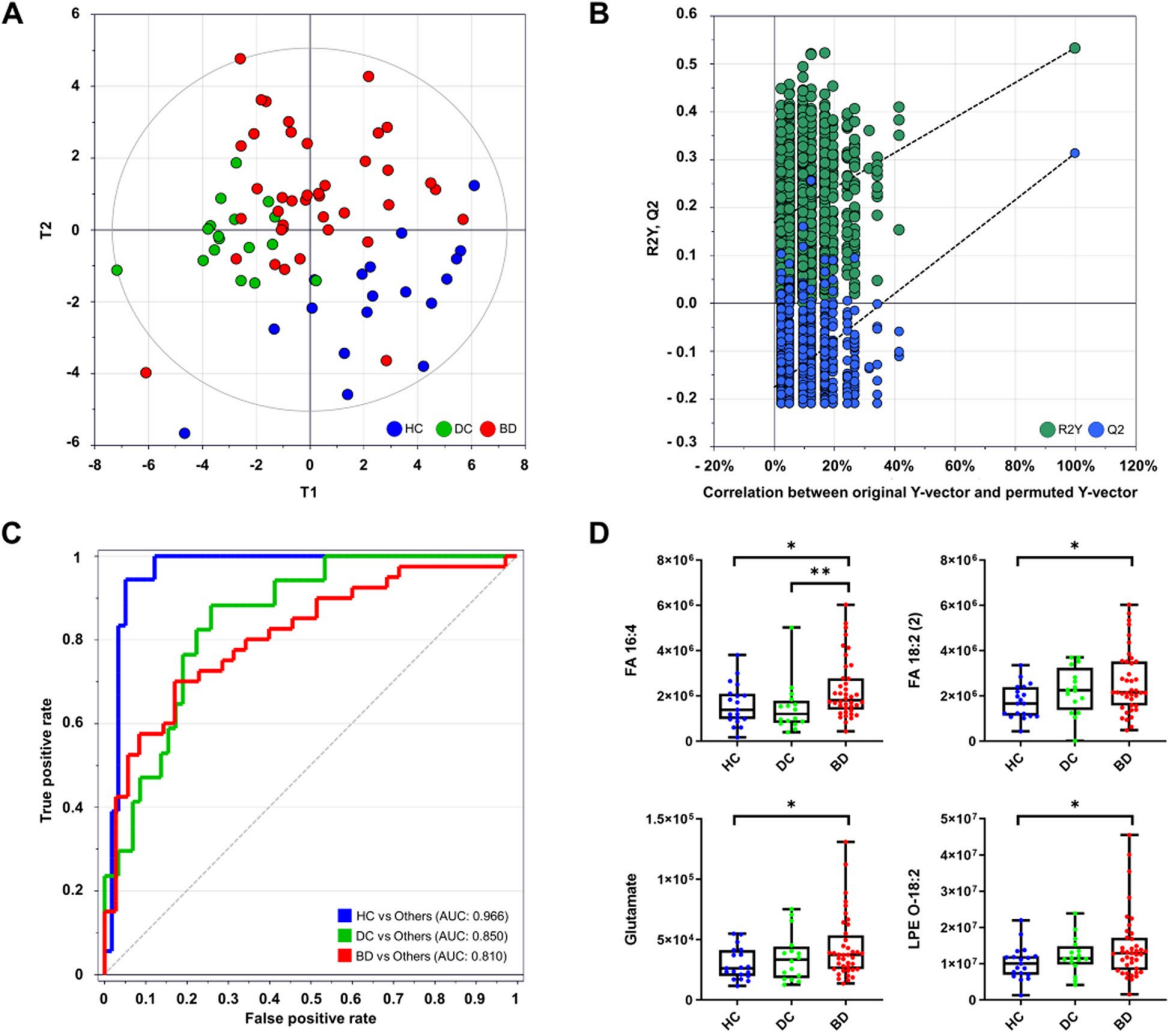
Identification of BD metabolic biomarker in plasma. **A** Score scatter plot of PLS-DA for BD with HC and DC. **B** 999 times permutation plot for PLS-DA (**C**) ROC analysis. AUC values of 13 metabolites’ composite ranged from 0.810 to 0.966. **D** Box and whisker plots presented relative abundances of BD-unique metabolites. The box indicates the interquartile range, and the whisker indicates the minimum and maximum values for all data (Mann-Whitney test, *p < 0.05, **p < 0.01)

We further explored biomarker candidates for BD against HC and DC. Partial least squares-discriminant analysis (PLS-DA) was performed to evaluate the covariance and correlation structures of the integrative metabolic profiles (Fig. 2A). Only significantly altered compounds were used for the analysis (*p* < 0.05, Mann-Whitney test). The resultant scatter plot showed a moderate level of discrimination between the three groups (R2Y = 0.369, Q2Y = 0.188). The model was validated using a 999-permutation test and CV-ANOVA (analysis of variance testing of cross-validated predictive residuals), which demonstrated a suitable model power as manifested by *y*-intercepts, slopes, and the significance level (*p* < 0.01), respectively (Fig. 2B).

To construct a universal biomarker panel exclusively for BD against HC and DC, we prioritized the metabolites using variable importance projection (VIP) based on the PLS-DA model. The diagnostic accuracy of the biomarker panel was evaluated using receiver operating characteristic (ROC) analysis. Thirteen metabolites were selected, considering the diagnostic accuracy and minimal number of components. The panel consisted of FA 20:4;2O (1), FA 20:4;2O (2), FA 16:4, sphingosine 1-phosphate, linolenic acid, citric acid, glucose, FA 16:4;2O, 1-monostearin, pyrophosphate, glutamate, xylose, and LPE O-18:2. The area under the curve (AUC) values were above 0.8 for all comparisons (BD, HC, and DC) (Fig. 2C). The AUC was 0.966 (95% confidence interval, 0.896–0.994) for HC compared with the others. The AUCs were 0.810 (0.704–0.892) with 70.00 sensitivity and 82.86 specificity (*p* < 0.0001) and 0.851 (0.750–0.923) for BD and DC, respectively. Among the selected biomarkers, FA 16:4, FA 18:2, glutamate, and LPE O-18:2 were specific to BD (Fig. 2D).

### Ether-linked lysophosphatidylethanolamine and lysophosphatidylcholine metabolism were altered in PBMCs of BD patients

**Fig. 3:**
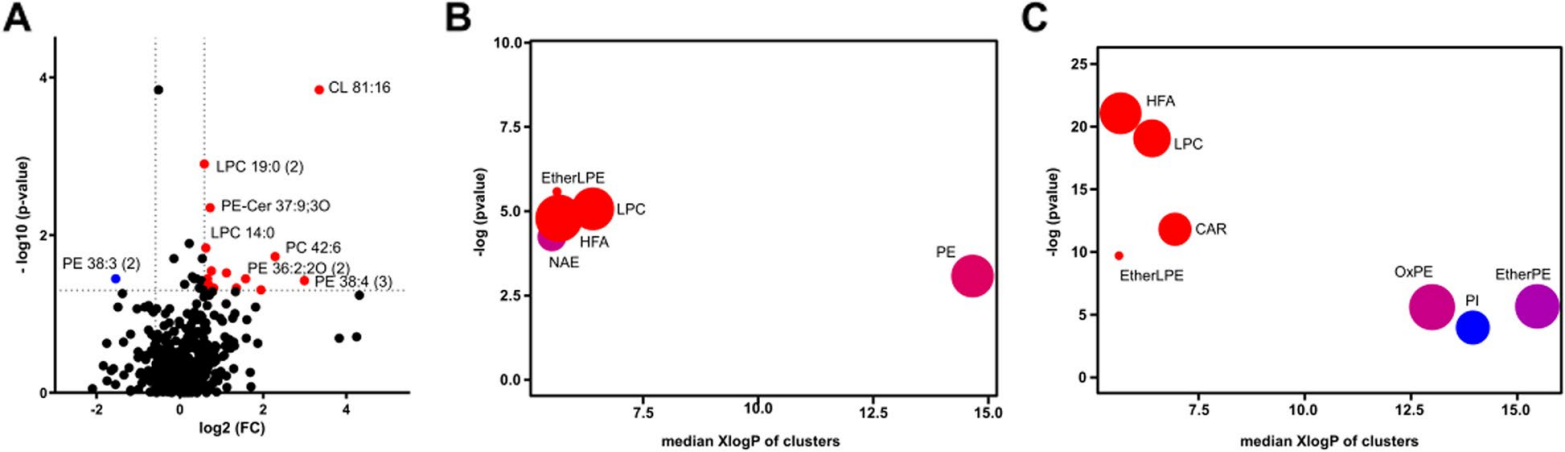
Characteristics of PBMC lipidome in BD. **A** Volcano plot for compounds altered in BD compared to HC. The *p*-value is calculated based on the Mann-Whitney test. Red and blue indicate a 1.5-fold increase and decrease, respectively, in BD. ChemRICH analysis of BD compared with HC (**B**) and DC (**C**). The node color scale represents the ratio of an increased (red) or decreased (blue) compound. The node size means the total number of lipids in each cluster

We performed untargeted lipid profiling of PBMCs, which resulted in the structural annotation of 345 lipids. The most remarkable difference was found in cardiolipin (CL) 81:16, which showed a more than 10-fold increase in BD compared to that in HC (Fig. 3A).

At the chemical class level, the EtherLPE, lysophosphatidylcholine (LPC), and HFA clusters showed significant differences between the HC and BD groups (Fig. 3B). EtherLPEs are substantially upregulated in BD patients (LPE O-16:1, LPE O-18:1, and LPE O-18:2). Five LPCs were significantly upregulated in BD patients, including LPC 14:0, LPC 15:0, LPC 16:0, LPC 19:0, and LPC 20:1 (Table S2). Dysregulation of EtherLPE, LPC, and HFA was common in the BD and DC groups, whereas PE and N-acylethanolamine (NAE) were specific to the BD group (Fig. 3B, C). PE 36:2, PE 36:2;O, and PE 36:2;2O showed BD-specific upregulation (Fig. S3). NAE 7:0 was significantly higher in BD patients than in HCs and DCs (Table S2).

**Fig. 4:**
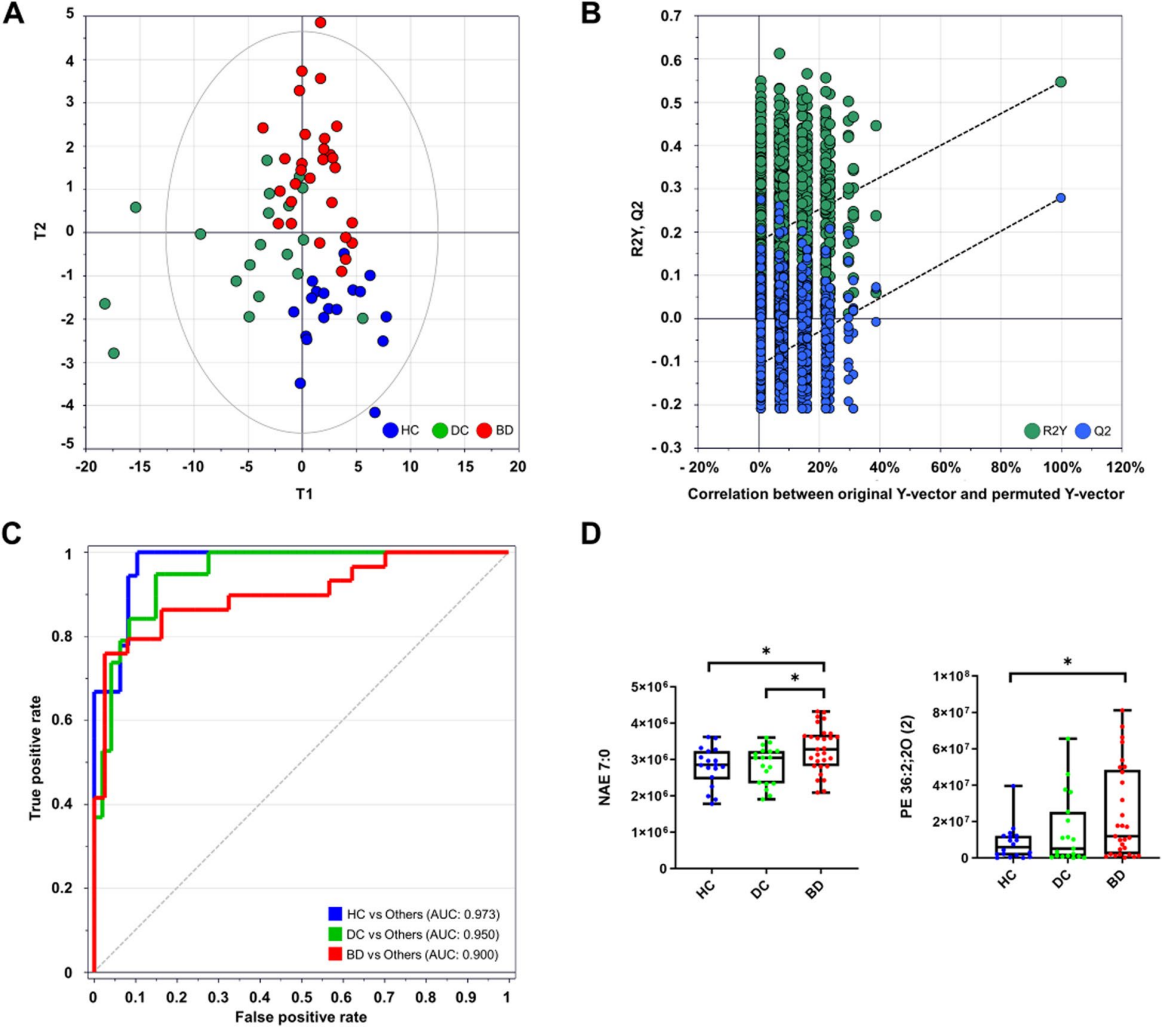
Discovery of unique PBMC biomarkers in BD. **A** Score scatter plot of PLS-DA for BD with HC and DC. **B** 999 times permutation plot for PLS-DA (**C**) ROC analysis. AUC values of 10 lipid composites ranged from 0.900 to 0.950. **D** Box and whisker plots presented relative abundances of BD-unique lipids. The box indicates the interquartile range, and the whisker indicates the minimum and maximum values for all data (Mann-Whitney test, *p < 0.05)

The PLS-DA scatter plot showed a clear separation according to different groups (R2Y = 0.488, Q2Y = 0.233) (Fig. 4A). The permutation test demonstrated that the model power was acceptable, considering the slope and intercepts of the R2Y and Q2Y plots (Fig. 4B). VIP analysis prioritized ten lipids as a biomarker panel. The selected lipids are as follows: NAE 12:0, NAE 7:0, PE-Cer 37:9;3O, PE 36:2;2O, LPC 19:0, LPC 14:0, PE 38:4, PC 35:6, PG 40:7, and FA 20:5. Predictive performance was evaluated using AUC values of 0.950 (0.763–0.913) for BD. The AUCs were greater than 0.9 for all comparisons (Fig. 4C). Among the biomarkers, NAE 7:0 and PE 36:2-2O showed BD-specific patterns that distinguished them from DC (Fig. 4D).

We further investigated the common metabolic changes between plasma and PBMCs. A total of 25 lipids were concomitantly determined in both plasma and PBMCs. Among the 25 lipids, we identified 13 with similar patterns in BD compared to those in HC (Table S3). LPE O-18:2 was the only metabolite that was significantly modulated in both the plasma and PBMCs. LPE O-16:1 and LPE O-18:1 also increased in PBMCs, but the changes were not significant. S1P showed a twofold increase in both plasma and PBMCs in BD.

### S1P and EtherLPE were involved in disease activity-dependent metabolic reprogramming

**Fig. 5:**
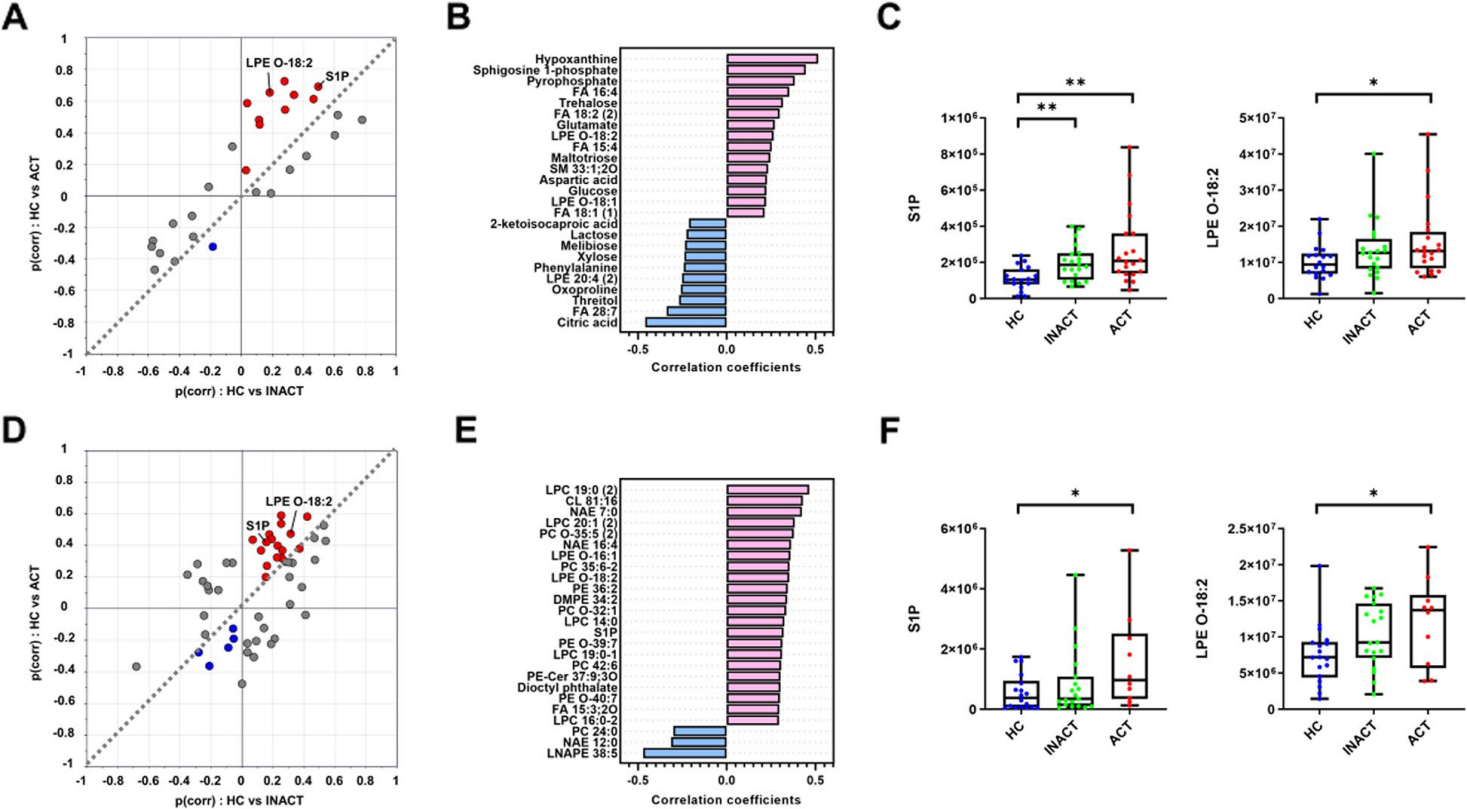
Metabolic signature according to disease activity. OPLS-based shared and unique structure (SUS) plot with *p* (corr) values of plasma (**A**) and PBMCs (**D**). The compounds in the upper region (red) showed a gradual increase with stage transition. Spearman rank analysis identified metabolites with patterns corresponding to the remission-relapse transition in plasma (**B**) and PBMCs (**E**). Box and whisker plots show compounds with upregulation in both remission and relapse compared to HC in plasma (**C**) and PBMCs (**F**) (Mann-Whitney test, *p < 0.05, **p < 0.01)

To identify disease activity-associated metabolites (e.g., relapse vs. remission), a shared-and-unique-structures (SUS) plot analysis was performed using the OPLS model (Fig. 5A, C). Multivariate statistics detect common and unique features given multiple pairwise comparisons with common references (e.g., HC-BD active vs. HC-BD inactive). Disease-status-specific metabolites were screened, and their patterns were recapitulated using Spearman correlation analysis. A common activity association was found in S1P and LPE O-18:2 in plasma and PBMCs. S1P and LPE O-18:2 showed a significant correlation with disease activity in plasma and PBMCs (Fig. 5B, D). In the plasma, FA 18:2, FA 16:4, hypoxanthine, pyrophosphate, trehalose, and glutamate were positively correlated, whereas citric acid, FA 28:7, and threitol showed inverse correlations. Active status was characterized by a gradual increase in various types of PBMC lipids, including LPC, PE, and NAE.

Furthermore, a confounding effect of clinical phenotype was evaluated by partial correlation analysis following Fisher’s exact test and permutational multivariate analysis of variance (PERMANOVA). In plasma, the clinical phenotype was not different by disease activity (Table S4). In PBMC, uveitis presented a significant difference between the active and inactive groups. PERMANOVA showed the significant effect of uveitis on the PBMC metabolome (R2 = 0.06, *p*-value = 0.018). Consequently, partial correlation analysis was applied to identify disease activity-associated metabolites, controlling for uveitis. A total of 23 compounds were significantly associated with disease activity, including S1P and LPE O-18:2 (Table S4).

We sought a potential association between metabolites and clinical manifestations or laboratory data in BD and DC groups. Less than 10% of the incidence was excluded in analysis. PERMANOVA calculated the explained variance of the clinical manifestations and laboratory tests for metabolome. The list of clinical factors with significant explained variance was genital ulcer in plasma and genital ulcer and ANA antibody in PBMC (Figure S4A). Subsequently, multiple linear regression identified the metabolites which were correlated with genital ulcer and ANA antibody and were independent of disease group (BD and DC) (Figure S4B). In the plasma, glycolic acid, serine, FA 14:0, and FA 28:7 were positively correlated with genital ulcers, whereas ethanolamine, tagatose, and fructose were negatively correlated. In PBMC, the alteration of PE is associated with genital ulcers and ANA antibody. Five phosphocholines show significant association with ANA antibody in PBMC.

## Discussion

Metabolomics is a promising technology for clinical applications, including biomarkers for the diagnosis and prognosis of diseases. Previous studies have applied metabolomics to identify biomarkers of BD in various types of biological samples, such as serum, urine, and synovial fluid. Nonetheless, the clinical applicability and pathomechanism remain unclear, particularly due to the manifestations of BD similar to those of other diseases. Our current study characterized metabolic signatures with BD specificity, discovered differential biomarkers against HC and DC, and discriminated between active and inactive status based on integrative metabolomic profiles of blood plasma and PBMCs.

Our primary finding in the plasma metabolite profiles is the perturbation of carbohydrate metabolism. BD patients showed significant alterations in glucose, fructose, tagatose, xylose, myo-inositol, and threitol levels. Dysregulation of carbohydrates and sugar alcohols is associated with inflammation and insulin resistance in BD [25, 26]. Increased levels of cytokines in BD, including tumor necrosis factor-alpha (TNF-α), interferon (IFN), and interleukin (IL)-1, have been shown to affect glucose homeostasis [27, 28]. Citrate, a TCA intermediate, showed a significant change in BD, whereas other intermediates showed marginal differences. The exclusive alteration of citrate may be related to the dysregulation of mitochondrial citrate carrier (CIC) and ATP citrate lyase (ACLY), which were previously evaluated in BD patients [29]. The study proposed that the upregulation of these genes causes overproduction of prostaglandin, reactive oxygen species (ROS), and nitric oxide (NO).

The second distinct feature was the exclusive increase in PUFA and linoleic acid in BD patients, but not in DCs. A significant increase in PUFA has been reported to induce ROS production in human lymphocytes [30]. Another PUFA (FA 16:4) showed specific alterations in BD patients. FA 16:4 has been shown to activate splenic macrophages [31]. Similarly, the oxidized forms of PUFAs, eicosanoids (FA 20:4;2O, FA 20:4;3O), differed significantly in both BD and DC, relative to HC. Eicosanoids have been proposed to have proinflammatory and immunoregulatory activities implicated in autoimmune and allergic diseases [32, 33].

Dysregulation of FAs coincides with the characteristic alteration of phospholipids. Cellular lipid homeostasis plays a significant role in cytokine production by innate immune cells and antibody production by adaptive immune cells. The pathogenesis of autoimmune disorders, BD, is affected by dysregulated lipid metabolism. Sphingosine 1-phosphate (S1P) and ether-linked lysophosphatidylethanolamine (EtherLPE) were upregulated in the BD group. S1P is associated with inflammatory signaling and immune cell trafficking [34]. Elevated S1P levels have been reported in various autoimmune diseases, including multiple sclerosis, rheumatoid arthritis, inflammatory bowel disease, and systemic lupus erythematosus [35]. Similarly, we identified elevated EtherLPEs specific to BD patients as follows: LPE O-18:2 (*p* = 0.049), LPE O-18:1 (*p* = 0.091), and LPE O-16:1 (*p* = 0.088). Ether-linked phospholipids play a critical role in immune cell differentiation [36], which may involve the potential hyperactivity of immunity in BD.

PBMCs are a mixture of immune cells comprising T cells, B cells, natural killer cells, monocytes, and dendritic cells, indicating immune homeostasis [37]. Dysregulation of PBMCs in BD patients has been linked to proinflammatory cytokines and immunomodulation [38, 39]. Accordingly, we examined the response of PBMC metabolism to BD pathology. The primary feature was the dysregulation of lysophosphatidylcholine (LPC) and EtherLPE. Five LPCs were significantly upregulated in BD patients, including LPC 14:0, LPC 15:0, LPC 16:0, LPC 19:0, and LPC 20:1. LPCs are involved in inflammation, hemostasis, and cytotoxicity. Phospholipids are produced from the cell membrane-derived phosphatidylcholine (PC) by phospholipase A2 (PLA2). An overexpression of PLA2 has been reported in BD patients. Macrophages in BD patients actively secrete PLA2. As PLA2 is secreted, proinflammatory products, such as LPC and oxidized non-esterified FAs, are produced. LPC production is also mediated by platelet-activating factor (PAF) receptors. PAF are produced from membrane-derived lipids by inflammatory stimulation with TNF-α and IL-1. PAF regulates various biological responses, including degranulation of blood cells, vascular permeability, and hypotension [40]. LPC overproduction in BD may be related to the upregulation of PAF, as previously reported [41]. EtherLPEs, such as LPE O-16:1, LPE O-18:1, and LPE O-18:2, were upregulated in BD. The upregulation of EtherLPE also induces PAF synthesis in human leukocytes [40].

Cardiolipin (CL) and n-acyl ethanolamine (NAE) levels significantly increased. CL 81:16 showed the highest increase in BD, but not in DC, compared with that in HC. CL is related to the thrombotic response. Destruction by anticardiolipin antibodies leads to the formation of a blood clot. Anticardiolipin antibodies have been extensively studied as a clinical manifestation of BD [42]. NAE7:0 is an FA amide involved in innate immunity, cell protection, and inflammation modulation [43]. Phosphatidylethanolamine (PE) showed an overall increase, of which PE 36:2 and its oxidized forms (PE 36:2;O and PE 36:2;2O) showed the most significant increase in BD. PE 36:2 has been implicated in the membrane phenotypes of macrophages, which are determined by the immune response [44]. Oxidized PEs have been reported to be associated with the PAF biosynthesis pathway and blood pressure regulation [45]. Oxidized phospholipids have shown different biological activities (e.g., regulation of cellular and immune signaling) from non-oxidized forms [46].

The course of BD is characterized by a repeated period of remission and activation, which is unpredictable. For proper treatment, the patient’s condition must be accurately diagnosed. Thus, we comparatively analyzed the metabolic differences between the active and the inactive states in the plasma and PBMCs. In both plasma and PBMCs, gradual increases were observed in S1P and LPE O-18:2. S1P is produced by sphingosine kinases (SKs), which are activated by cytokines, such as TNF-α [12]. Increased levels of inflammatory mediators have been reported in BD patients. In addition, plasma S1P affects lymphocyte egress and lymphatic patterning, which may be a consequence of an interaction between the blood and the PBMCs [47, 48]. LPE O-18:2 is a plasmalogen and a proinflammatory lysophospholipid [49]. PUFAs were correlated with disease activity in plasma samples, including FA 15:4, FA 16:4, FA 18:2, FA 20:4, 4O, and FA 28,7. PUFAs provide fatty acyl for eicosanoid biosynthesis, which mediates proinflammatory signaling and vascular responses [50]. In PBMC samples, LPC and NAE increased gradually according to disease activity, which is associated with the innate immune response [51, 52].

This study has several limitations, including the relatively small sample size and single medical cohort. For the practical application of biomarkers, potential biomarkers should be validated and reproducible in larger sample sizes using independent validation cohorts.

## Conclusion

In the current study, we explored the unique metabolic regulation of BD in plasma and PBMCs based on high-throughput untargeted metabolic profiling. Accordingly, we proposed a comprehensive BD mechanism and discovered a biomarker panel for differential diagnosis. S1P and EtherLPE 18:2 showed a common association with disease status in plasma and PBMCs. This study may help to understand the disease mechanism and suggest a key molecule for biochemical characterization according to disease activity.

## Supplementary Information

**Additional file 1.** Supplementary figures and tables.

## Data Availability

The datasets used and/or analyzed during the current study are available from the corresponding author on request.

## Abbreviations

BD: Behçet’s disease
PBMCs: Peripheral blood mononuclear cells
GC-TOF MS: Gas chromatography time-of-flight mass spectrometry
UPLC-Orbitrap MS: Ultraperformance liquid chromatography Orbitrap mass spectrometry
IL: Interleukin
TNF-α: Tumor necrosis factor-alpha
PFAPA: Periodic fever, aphthous stomatitis, pharyngitis, adenitis
ESR: Erythrocyte sedimentation rate
CRP: C-reactive protein
RAU: Recurrent aphthous ulcer
DCs: Disease controls (patients with similar symptoms)
HCs: Healthy controls
PLS-DA: Partial least squares-discriminant analysis
VIP: Variable importance in projection
ROC: Receiver operating characteristic
PCA: Principal component analysis
PE: Phosphatidylethanolamine
HFA: Hydroxy fatty acids
PUFAs: Polyunsaturated fatty acids
S1P: Sphingosine 1-phosphate
FA 18:2: Linolenic acid
OxPE: Oxidized phosphatidylethanolamine
CV-ANOVA: Analysis of variance testing of cross-validated predictive residuals
AUC: Area under the curve
CL: Cardiolipin
LPC: Lysophosphatidylcholine
NAE: N-Acylethanolamine
EtherLPE: Ether-linked lysophosphatidylethanolamine
PC: Phosphatidylcholine
PLA: Phospholipase
PAF: Platelet-activating factor
