# Supplementary material for: Integrative metabolomics of plasma and PBMCs identifies distinctive metabolic signatures in Behçet’s disease
Source: Arthritis Res Ther. 2023 Jan 7;25:5. doi: 10.1186/s13075-022-02986-5 (PMC9824930; doi:10.1186/s13075-022-02986-5)
Supplement: Supplementary file 1 — Additional file 1. Supplementary figures and tables. [file 13075_2022_2986_MOESM1_ESM.pptx]

## Slide 1
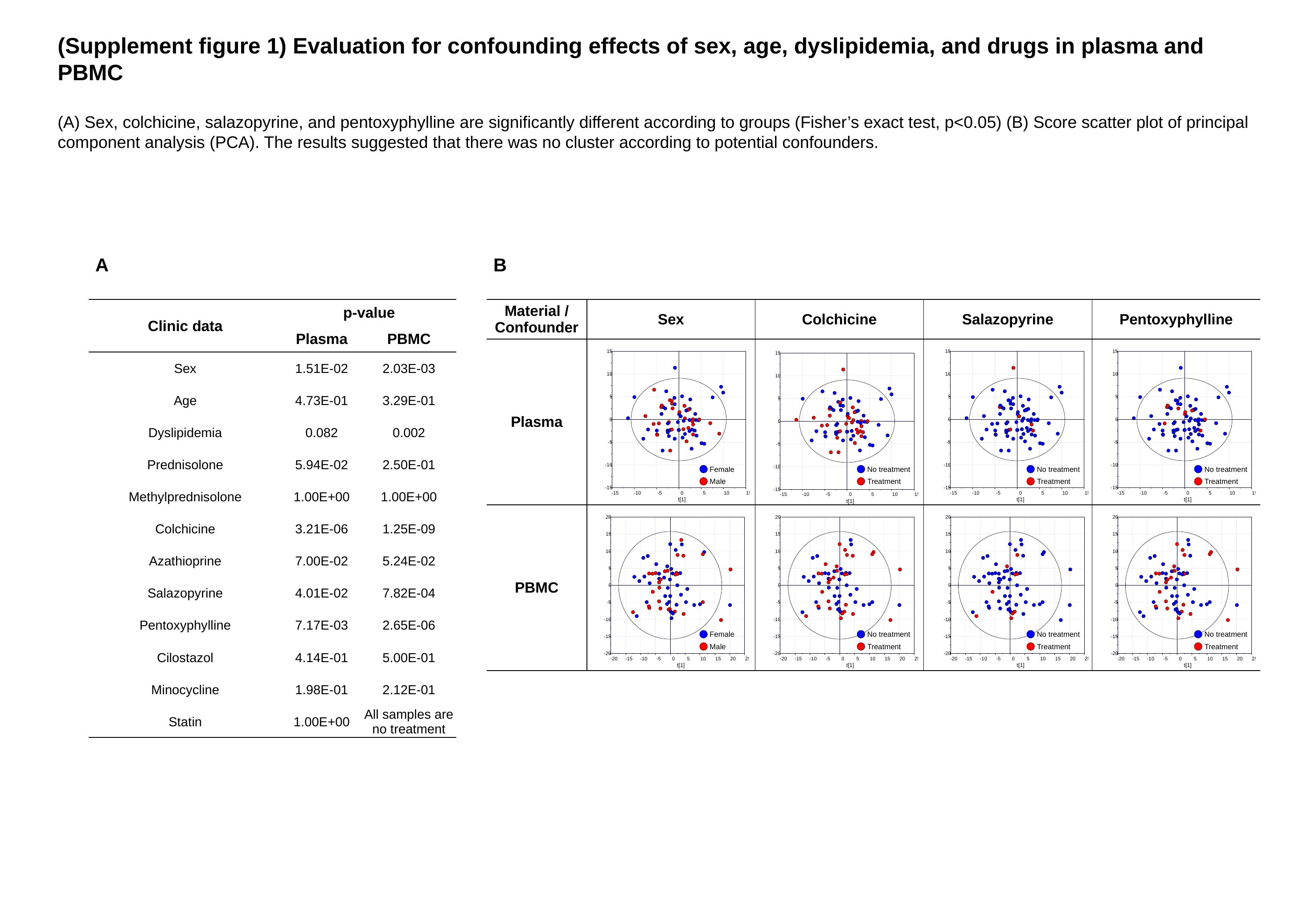

(Supplement figure 1) Evaluation for confounding effects of sex, age, dyslipidemia, and drugs in plasma and PBMC
(A) Sex, colchicine, salazopyrine, and pentoxyphylline are significantly different according to groups (Fisher’s exact test, p<0.05) (B) Score scatter plot of principal component analysis (PCA). The results suggested that there was no cluster according to potential confounders.
A
B
| Clinic data | p-value | PBMC |
| --- | --- | --- |
| | Plasma | PBMC |
| Sex | 1.51E-02 | 2.03E-03 |
| Age | 4.73E-01 | 3.29E-01 |
| Dyslipidemia | 0.082 | 0.002 |
| Prednisolone | 5.94E-02 | 2.50E-01 |
| Methylprednisolone | 1.00E+00 | 1.00E+00 |
| Colchicine | 3.21E-06 | 1.25E-09 |
| Azathioprine | 7.00E-02 | 5.24E-02 |
| Salazopyrine | 4.01E-02 | 7.82E-04 |
| Pentoxyphylline | 7.17E-03 | 2.65E-06 |
| Cilostazol | 4.14E-01 | 5.00E-01 |
| Minocycline | 1.98E-01 | 2.12E-01 |
| Statin | 1.00E+00 | All samples are no treatment |
| Material / Confounder | Sex | Colchicine | Salazopyrine | Pentoxyphylline |
| --- | --- | --- | --- | --- |
| Plasma | | | | |
| PBMC | | | | |
Female
No treatment
No treatment
No treatment
Male
Treatment
Treatment
Treatment
Female
No treatment
No treatment
No treatment
Male
Treatment
Treatment
Treatment

## Slide 2
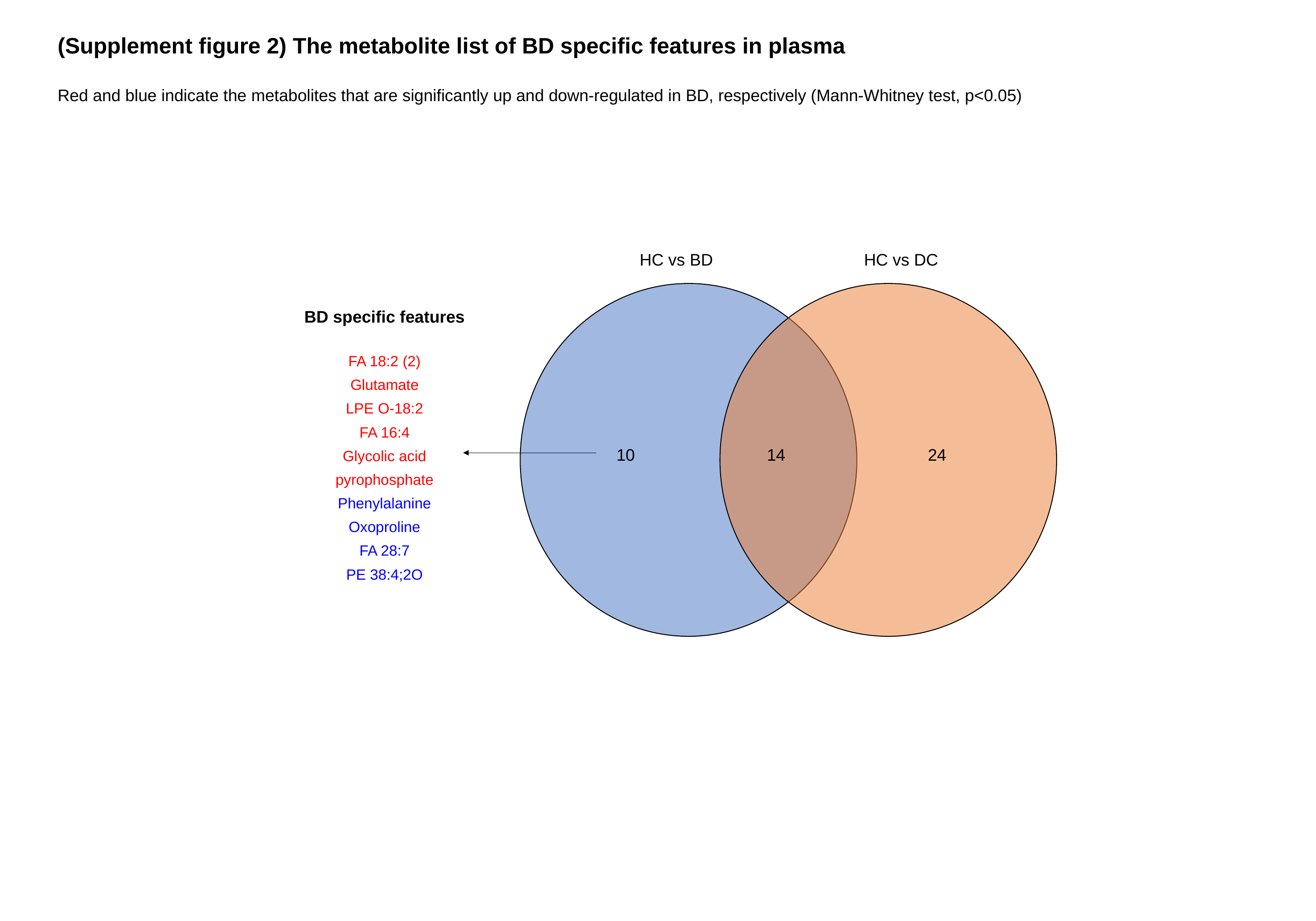

(Supplement figure 2) The metabolite list of BD specific features in plasma
Red and blue indicate the metabolites that are significantly up and down-regulated in BD, respectively (Mann-Whitney test, p<0.05)
HC vs BD
HC vs DC
BD specific features
10
14
24
| FA 18:2 (2) |
| --- |
| Glutamate |
| LPE O-18:2 |
| FA 16:4 |
| Glycolic acid |
| pyrophosphate |
| Phenylalanine |
| Oxoproline |
| FA 28:7 |
| PE 38:4;2O |

## Slide 3
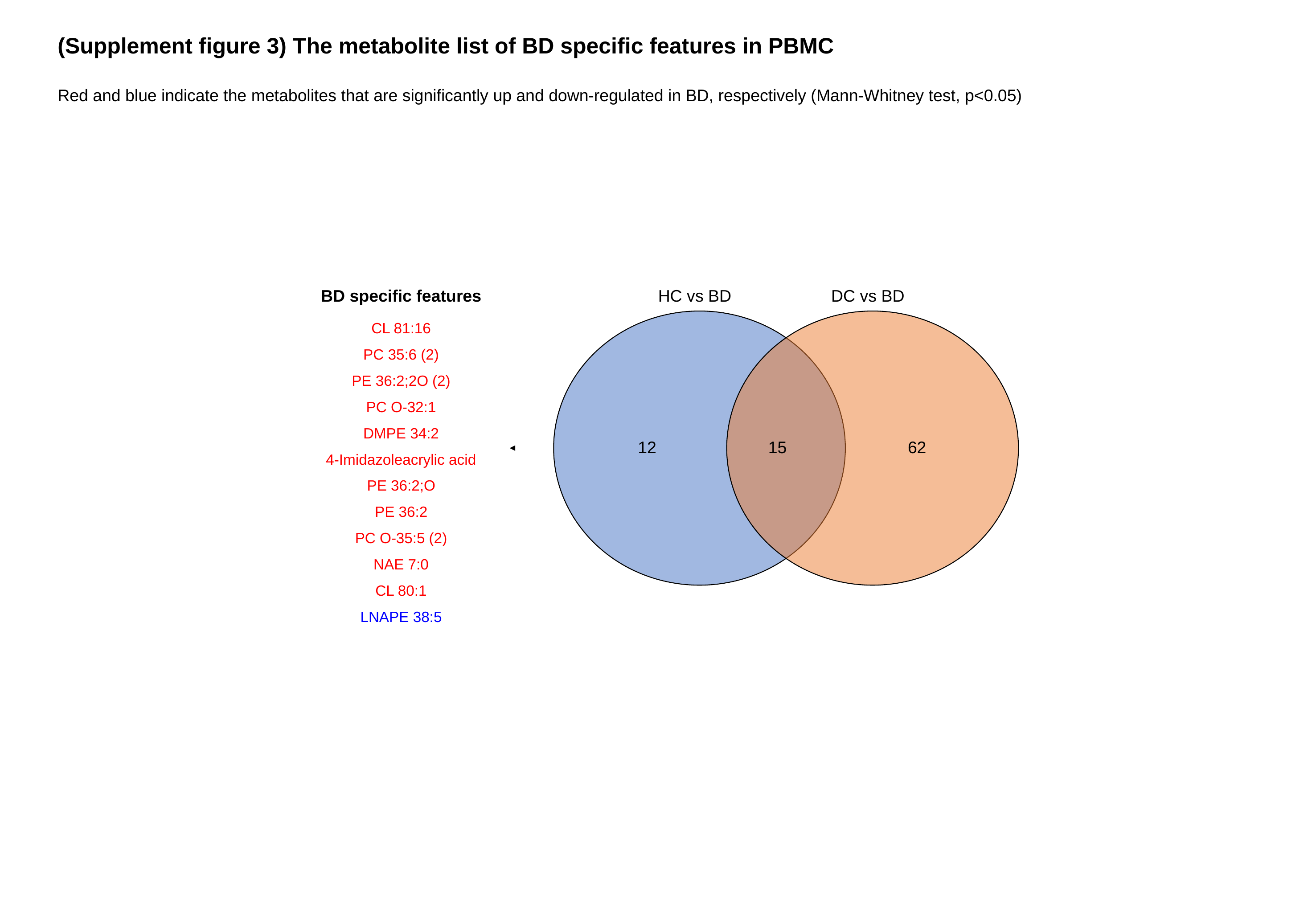

(Supplement figure 3) The metabolite list of BD specific features in PBMC
Red and blue indicate the metabolites that are significantly up and down-regulated in BD, respectively (Mann-Whitney test, p<0.05)
BD specific features
HC vs BD
DC vs BD
12
15
62
| CL 81:16 |
| --- |
| PC 35:6 (2) |
| PE 36:2;2O (2) |
| PC O-32:1 |
| DMPE 34:2 |
| 4-Imidazoleacrylic acid |
| PE 36:2;O |
| PE 36:2 |
| PC O-35:5 (2) |
| NAE 7:0 |
| CL 80:1 |
| LNAPE 38:5 |

## Slide 4
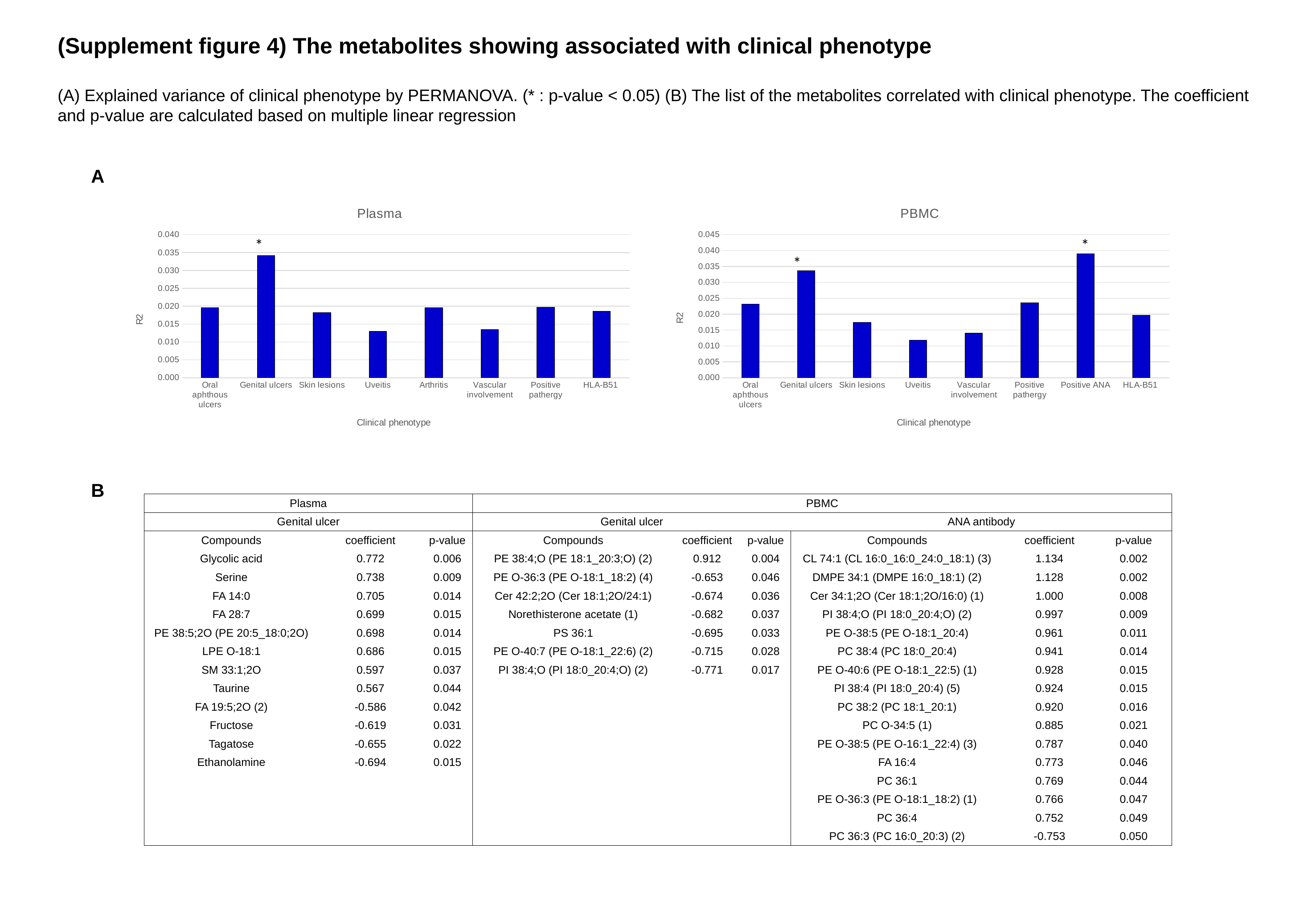

(Supplement figure 4) The metabolites showing associated with clinical phenotype
(A) Explained variance of clinical phenotype by PERMANOVA. (* : p-value < 0.05) (B) The list of the metabolites correlated with clinical phenotype. The coefficient and p-value are calculated based on multiple linear regression
A
### Chart: Plasma
| Category | R2 |
|---|---|
| Oral aphthous ulcers | 0.0196137320295511 |
| Genital ulcers | 0.0341209833456369 |
| Skin lesions | 0.0182170325367825 |
| Uveitis | 0.0130413084347089 |
| Arthritis | 0.0196344928181617 |
| Vascular involvement | 0.0135228759232648 |
| Positive pathergy | 0.0196810795619478 |
| HLA-B51 | 0.0185632072244942 |
### Chart: PBMC
| Category | R2 |
|---|---|
| Oral aphthous ulcers | 0.0231554125806331 |
| Genital ulcers | 0.0336200383033186 |
| Skin lesions | 0.0174195182083767 |
| Uveitis | 0.0117740399927043 |
| Vascular involvement | 0.0140908168690064 |
| Positive pathergy | 0.0236463657405249 |
| Positive ANA | 0.0389968189779339 |
| HLA-B51 | 0.019647516295281 |*
*
*
B
| Plasma | | | PBMC | | | | | |
| --- | --- | --- | --- | --- | --- | --- | --- | --- |
| Genital ulcer | | | Genital ulcer | | | ANA antibody | | |
| Compounds | coefficient | p-value | Compounds | coefficient | p-value | Compounds | coefficient | p-value |
| Glycolic acid | 0.772 | 0.006 | PE 38:4;O (PE 18:1\_20:3;O) (2) | 0.912 | 0.004 | CL 74:1 (CL 16:0\_16:0\_24:0\_18:1) (3) | 1.134 | 0.002 |
| Serine | 0.738 | 0.009 | PE O-36:3 (PE O-18:1\_18:2) (4) | -0.653 | 0.046 | DMPE 34:1 (DMPE 16:0\_18:1) (2) | 1.128 | 0.002 |
| FA 14:0 | 0.705 | 0.014 | Cer 42:2;2O (Cer 18:1;2O/24:1) | -0.674 | 0.036 | Cer 34:1;2O (Cer 18:1;2O/16:0) (1) | 1.000 | 0.008 |
| FA 28:7 | 0.699 | 0.015 | Norethisterone acetate (1) | -0.682 | 0.037 | PI 38:4;O (PI 18:0\_20:4;O) (2) | 0.997 | 0.009 |
| PE 38:5;2O (PE 20:5\_18:0;2O) | 0.698 | 0.014 | PS 36:1 | -0.695 | 0.033 | PE O-38:5 (PE O-18:1\_20:4) | 0.961 | 0.011 |
| LPE O-18:1 | 0.686 | 0.015 | PE O-40:7 (PE O-18:1\_22:6) (2) | -0.715 | 0.028 | PC 38:4 (PC 18:0\_20:4) | 0.941 | 0.014 |
| SM 33:1;2O | 0.597 | 0.037 | PI 38:4;O (PI 18:0\_20:4;O) (2) | -0.771 | 0.017 | PE O-40:6 (PE O-18:1\_22:5) (1) | 0.928 | 0.015 |
| Taurine | 0.567 | 0.044 | | | | PI 38:4 (PI 18:0\_20:4) (5) | 0.924 | 0.015 |
| FA 19:5;2O (2) | -0.586 | 0.042 | | | | PC 38:2 (PC 18:1\_20:1) | 0.920 | 0.016 |
| Fructose | -0.619 | 0.031 | | | | PC O-34:5 (1) | 0.885 | 0.021 |
| Tagatose | -0.655 | 0.022 | | | | PE O-38:5 (PE O-16:1\_22:4) (3) | 0.787 | 0.040 |
| Ethanolamine | -0.694 | 0.015 | | | | FA 16:4 | 0.773 | 0.046 |
| | | | | | | PC 36:1 | 0.769 | 0.044 |
| | | | | | | PE O-36:3 (PE O-18:1\_18:2) (1) | 0.766 | 0.047 |
| | | | | | | PC 36:4 | 0.752 | 0.049 |
| | | | | | | PC 36:3 (PC 16:0\_20:3) (2) | -0.753 | 0.050 |

## Slide 5
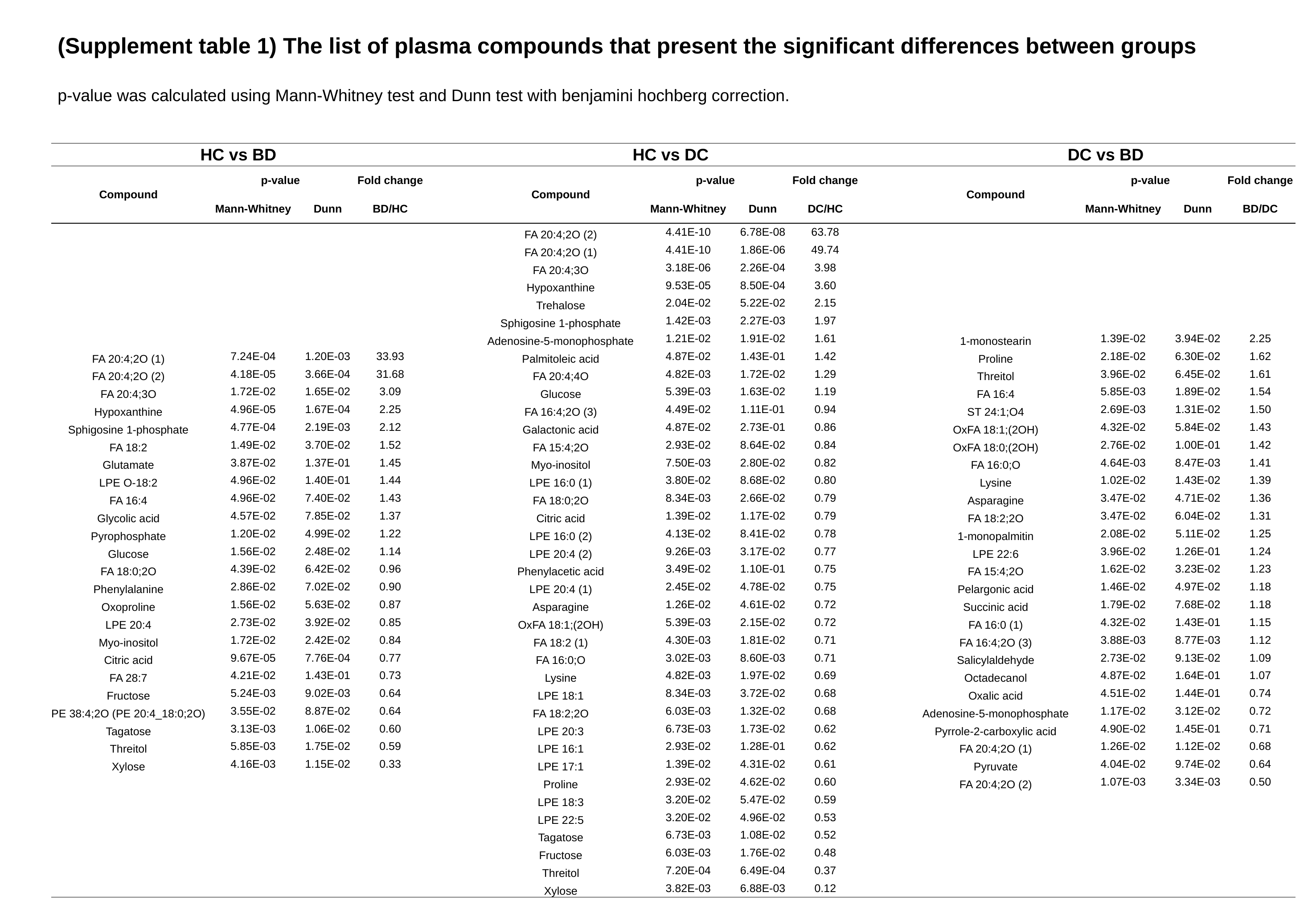

(Supplement table 1) The list of plasma compounds that present the significant differences between groups
p-value was calculated using Mann-Whitney test and Dunn test with benjamini hochberg correction.
| HC vs BD | | | | | HC vs DC | | | | | DC vs BD | | | |
| --- | --- | --- | --- | --- | --- | --- | --- | --- | --- | --- | --- | --- | --- |
| Compound | p-value | | Fold change | | Compound | p-value | | Fold change | | Compound | p-value | | Fold change |
| | Mann-Whitney | Dunn | BD/HC | | | Mann-Whitney | Dunn | DC/HC | | | Mann-Whitney | Dunn | BD/DC |
| | | | | | FA 20:4;2O (2) | 4.41E-10 | 6.78E-08 | 63.78 | | | | | |
| | | | | | FA 20:4;2O (1) | 4.41E-10 | 1.86E-06 | 49.74 | | | | | |
| | | | | | FA 20:4;3O | 3.18E-06 | 2.26E-04 | 3.98 | | | | | |
| | | | | | Hypoxanthine | 9.53E-05 | 8.50E-04 | 3.60 | | | | | |
| | | | | | Trehalose | 2.04E-02 | 5.22E-02 | 2.15 | | | | | |
| | | | | | Sphigosine 1-phosphate | 1.42E-03 | 2.27E-03 | 1.97 | | | | | |
| | | | | | Adenosine-5-monophosphate | 1.21E-02 | 1.91E-02 | 1.61 | | 1-monostearin | 1.39E-02 | 3.94E-02 | 2.25 |
| FA 20:4;2O (1) | 7.24E-04 | 1.20E-03 | 33.93 | | Palmitoleic acid | 4.87E-02 | 1.43E-01 | 1.42 | | Proline | 2.18E-02 | 6.30E-02 | 1.62 |
| FA 20:4;2O (2) | 4.18E-05 | 3.66E-04 | 31.68 | | FA 20:4;4O | 4.82E-03 | 1.72E-02 | 1.29 | | Threitol | 3.96E-02 | 6.45E-02 | 1.61 |
| FA 20:4;3O | 1.72E-02 | 1.65E-02 | 3.09 | | Glucose | 5.39E-03 | 1.63E-02 | 1.19 | | FA 16:4 | 5.85E-03 | 1.89E-02 | 1.54 |
| Hypoxanthine | 4.96E-05 | 1.67E-04 | 2.25 | | FA 16:4;2O (3) | 4.49E-02 | 1.11E-01 | 0.94 | | ST 24:1;O4 | 2.69E-03 | 1.31E-02 | 1.50 |
| Sphigosine 1-phosphate | 4.77E-04 | 2.19E-03 | 2.12 | | Galactonic acid | 4.87E-02 | 2.73E-01 | 0.86 | | OxFA 18:1;(2OH) | 4.32E-02 | 5.84E-02 | 1.43 |
| FA 18:2 | 1.49E-02 | 3.70E-02 | 1.52 | | FA 15:4;2O | 2.93E-02 | 8.64E-02 | 0.84 | | OxFA 18:0;(2OH) | 2.76E-02 | 1.00E-01 | 1.42 |
| Glutamate | 3.87E-02 | 1.37E-01 | 1.45 | | Myo-inositol | 7.50E-03 | 2.80E-02 | 0.82 | | FA 16:0;O | 4.64E-03 | 8.47E-03 | 1.41 |
| LPE O-18:2 | 4.96E-02 | 1.40E-01 | 1.44 | | LPE 16:0 (1) | 3.80E-02 | 8.68E-02 | 0.80 | | Lysine | 1.02E-02 | 1.43E-02 | 1.39 |
| FA 16:4 | 4.96E-02 | 7.40E-02 | 1.43 | | FA 18:0;2O | 8.34E-03 | 2.66E-02 | 0.79 | | Asparagine | 3.47E-02 | 4.71E-02 | 1.36 |
| Glycolic acid | 4.57E-02 | 7.85E-02 | 1.37 | | Citric acid | 1.39E-02 | 1.17E-02 | 0.79 | | FA 18:2;2O | 3.47E-02 | 6.04E-02 | 1.31 |
| Pyrophosphate | 1.20E-02 | 4.99E-02 | 1.22 | | LPE 16:0 (2) | 4.13E-02 | 8.41E-02 | 0.78 | | 1-monopalmitin | 2.08E-02 | 5.11E-02 | 1.25 |
| Glucose | 1.56E-02 | 2.48E-02 | 1.14 | | LPE 20:4 (2) | 9.26E-03 | 3.17E-02 | 0.77 | | LPE 22:6 | 3.96E-02 | 1.26E-01 | 1.24 |
| FA 18:0;2O | 4.39E-02 | 6.42E-02 | 0.96 | | Phenylacetic acid | 3.49E-02 | 1.10E-01 | 0.75 | | FA 15:4;2O | 1.62E-02 | 3.23E-02 | 1.23 |
| Phenylalanine | 2.86E-02 | 7.02E-02 | 0.90 | | LPE 20:4 (1) | 2.45E-02 | 4.78E-02 | 0.75 | | Pelargonic acid | 1.46E-02 | 4.97E-02 | 1.18 |
| Oxoproline | 1.56E-02 | 5.63E-02 | 0.87 | | Asparagine | 1.26E-02 | 4.61E-02 | 0.72 | | Succinic acid | 1.79E-02 | 7.68E-02 | 1.18 |
| LPE 20:4 | 2.73E-02 | 3.92E-02 | 0.85 | | OxFA 18:1;(2OH) | 5.39E-03 | 2.15E-02 | 0.72 | | FA 16:0 (1) | 4.32E-02 | 1.43E-01 | 1.15 |
| Myo-inositol | 1.72E-02 | 2.42E-02 | 0.84 | | FA 18:2 (1) | 4.30E-03 | 1.81E-02 | 0.71 | | FA 16:4;2O (3) | 3.88E-03 | 8.77E-03 | 1.12 |
| Citric acid | 9.67E-05 | 7.76E-04 | 0.77 | | FA 16:0;O | 3.02E-03 | 8.60E-03 | 0.71 | | Salicylaldehyde | 2.73E-02 | 9.13E-02 | 1.09 |
| FA 28:7 | 4.21E-02 | 1.43E-01 | 0.73 | | Lysine | 4.82E-03 | 1.97E-02 | 0.69 | | Octadecanol | 4.87E-02 | 1.64E-01 | 1.07 |
| Fructose | 5.24E-03 | 9.02E-03 | 0.64 | | LPE 18:1 | 8.34E-03 | 3.72E-02 | 0.68 | | Oxalic acid | 4.51E-02 | 1.44E-01 | 0.74 |
| PE 38:4;2O (PE 20:4\_18:0;2O) | 3.55E-02 | 8.87E-02 | 0.64 | | FA 18:2;2O | 6.03E-03 | 1.32E-02 | 0.68 | | Adenosine-5-monophosphate | 1.17E-02 | 3.12E-02 | 0.72 |
| Tagatose | 3.13E-03 | 1.06E-02 | 0.60 | | LPE 20:3 | 6.73E-03 | 1.73E-02 | 0.62 | | Pyrrole-2-carboxylic acid | 4.90E-02 | 1.45E-01 | 0.71 |
| Threitol | 5.85E-03 | 1.75E-02 | 0.59 | | LPE 16:1 | 2.93E-02 | 1.28E-01 | 0.62 | | FA 20:4;2O (1) | 1.26E-02 | 1.12E-02 | 0.68 |
| Xylose | 4.16E-03 | 1.15E-02 | 0.33 | | LPE 17:1 | 1.39E-02 | 4.31E-02 | 0.61 | | Pyruvate | 4.04E-02 | 9.74E-02 | 0.64 |
| | | | | | Proline | 2.93E-02 | 4.62E-02 | 0.60 | | FA 20:4;2O (2) | 1.07E-03 | 3.34E-03 | 0.50 |
| | | | | | LPE 18:3 | 3.20E-02 | 5.47E-02 | 0.59 | | | | | |
| | | | | | LPE 22:5 | 3.20E-02 | 4.96E-02 | 0.53 | | | | | |
| | | | | | Tagatose | 6.73E-03 | 1.08E-02 | 0.52 | | | | | |
| | | | | | Fructose | 6.03E-03 | 1.76E-02 | 0.48 | | | | | |
| | | | | | Threitol | 7.20E-04 | 6.49E-04 | 0.37 | | | | | |
| | | | | | Xylose | 3.82E-03 | 6.88E-03 | 0.12 | | | | | |

## Slide 6
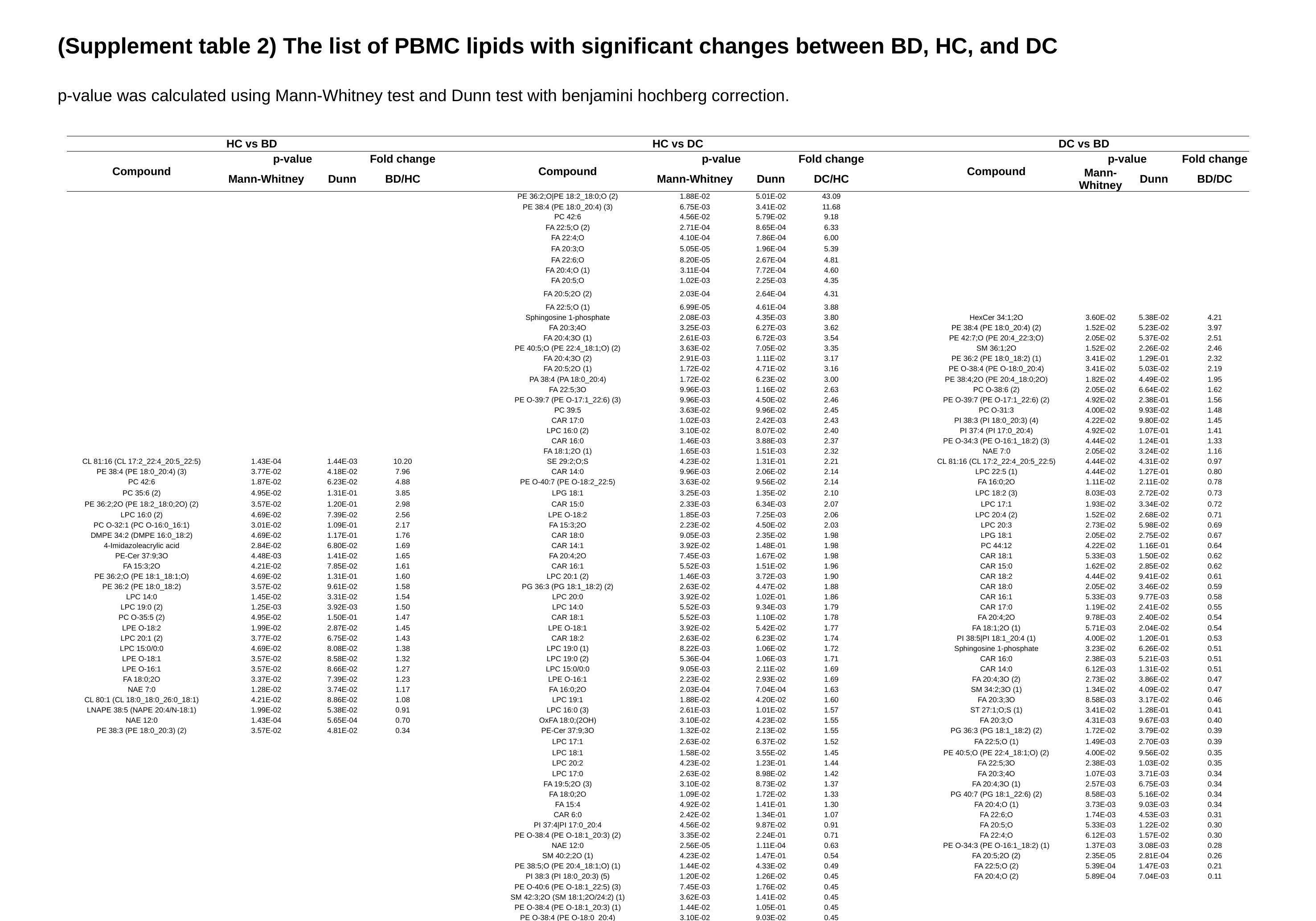

(Supplement table 2) The list of PBMC lipids with significant changes between BD, HC, and DC
p-value was calculated using Mann-Whitney test and Dunn test with benjamini hochberg correction.
| HC vs BD | | | | | HC vs DC | | | | | DC vs BD | | | |
| --- | --- | --- | --- | --- | --- | --- | --- | --- | --- | --- | --- | --- | --- |
| Compound | p-value | | Fold change | | Compound | p-value | | Fold change | | Compound | p-value | | Fold change |
| | Mann-Whitney | Dunn | BD/HC | | | Mann-Whitney | Dunn | DC/HC | | | Mann-Whitney | Dunn | BD/DC |
| | | | | | PE 36:2;O|PE 18:2\_18:0;O (2) | 1.88E-02 | 5.01E-02 | 43.09 | | | | | |
| | | | | | PE 38:4 (PE 18:0\_20:4) (3) | 6.75E-03 | 3.41E-02 | 11.68 | | | | | |
| | | | | | PC 42:6 | 4.56E-02 | 5.79E-02 | 9.18 | | | | | |
| | | | | | FA 22:5;O (2) | 2.71E-04 | 8.65E-04 | 6.33 | | | | | |
| | | | | | FA 22:4;O | 4.10E-04 | 7.86E-04 | 6.00 | | | | | |
| | | | | | FA 20:3;O | 5.05E-05 | 1.96E-04 | 5.39 | | | | | |
| | | | | | FA 22:6;O | 8.20E-05 | 2.67E-04 | 4.81 | | | | | |
| | | | | | FA 20:4;O (1) | 3.11E-04 | 7.72E-04 | 4.60 | | | | | |
| | | | | | FA 20:5;O | 1.02E-03 | 2.25E-03 | 4.35 | | | | | |
| | | | | | FA 20:5;2O (2) | 2.03E-04 | 2.64E-04 | 4.31 | | | | | |
| | | | | | FA 22:5;O (1) | 6.99E-05 | 4.61E-04 | 3.88 | | | | | |
| | | | | | Sphingosine 1-phosphate | 2.08E-03 | 4.35E-03 | 3.80 | | HexCer 34:1;2O | 3.60E-02 | 5.38E-02 | 4.21 |
| | | | | | FA 20:3;4O | 3.25E-03 | 6.27E-03 | 3.62 | | PE 38:4 (PE 18:0\_20:4) (2) | 1.52E-02 | 5.23E-02 | 3.97 |
| | | | | | FA 20:4;3O (1) | 2.61E-03 | 6.72E-03 | 3.54 | | PE 42:7;O (PE 20:4\_22:3;O) | 2.05E-02 | 5.37E-02 | 2.51 |
| | | | | | PE 40:5;O (PE 22:4\_18:1;O) (2) | 3.63E-02 | 7.05E-02 | 3.35 | | SM 36:1;2O | 1.52E-02 | 2.26E-02 | 2.46 |
| | | | | | FA 20:4;3O (2) | 2.91E-03 | 1.11E-02 | 3.17 | | PE 36:2 (PE 18:0\_18:2) (1) | 3.41E-02 | 1.29E-01 | 2.32 |
| | | | | | FA 20:5;2O (1) | 1.72E-02 | 4.71E-02 | 3.16 | | PE O-38:4 (PE O-18:0\_20:4) | 3.41E-02 | 5.03E-02 | 2.19 |
| | | | | | PA 38:4 (PA 18:0\_20:4) | 1.72E-02 | 6.23E-02 | 3.00 | | PE 38:4;2O (PE 20:4\_18:0;2O) | 1.82E-02 | 4.49E-02 | 1.95 |
| | | | | | FA 22:5;3O | 9.96E-03 | 1.16E-02 | 2.63 | | PC O-38:6 (2) | 2.05E-02 | 6.64E-02 | 1.62 |
| | | | | | PE O-39:7 (PE O-17:1\_22:6) (3) | 9.96E-03 | 4.50E-02 | 2.46 | | PE O-39:7 (PE O-17:1\_22:6) (2) | 4.92E-02 | 2.38E-01 | 1.56 |
| | | | | | PC 39:5 | 3.63E-02 | 9.96E-02 | 2.45 | | PC O-31:3 | 4.00E-02 | 9.93E-02 | 1.48 |
| | | | | | CAR 17:0 | 1.02E-03 | 2.42E-03 | 2.43 | | PI 38:3 (PI 18:0\_20:3) (4) | 4.22E-02 | 9.80E-02 | 1.45 |
| | | | | | LPC 16:0 (2) | 3.10E-02 | 8.07E-02 | 2.40 | | PI 37:4 (PI 17:0\_20:4) | 4.92E-02 | 1.07E-01 | 1.41 |
| | | | | | CAR 16:0 | 1.46E-03 | 3.88E-03 | 2.37 | | PE O-34:3 (PE O-16:1\_18:2) (3) | 4.44E-02 | 1.24E-01 | 1.33 |
| | | | | | FA 18:1;2O (1) | 1.65E-03 | 1.51E-03 | 2.32 | | NAE 7:0 | 2.05E-02 | 3.24E-02 | 1.16 |
| CL 81:16 (CL 17:2\_22:4\_20:5\_22:5) | 1.43E-04 | 1.44E-03 | 10.20 | | SE 29:2;O;S | 4.23E-02 | 1.31E-01 | 2.21 | | CL 81:16 (CL 17:2\_22:4\_20:5\_22:5) | 4.44E-02 | 4.31E-02 | 0.97 |
| PE 38:4 (PE 18:0\_20:4) (3) | 3.77E-02 | 4.18E-02 | 7.96 | | CAR 14:0 | 9.96E-03 | 2.06E-02 | 2.14 | | LPC 22:5 (1) | 4.44E-02 | 1.27E-01 | 0.80 |
| PC 42:6 | 1.87E-02 | 6.23E-02 | 4.88 | | PE O-40:7 (PE O-18:2\_22:5) | 3.63E-02 | 9.56E-02 | 2.14 | | FA 16:0;2O | 1.11E-02 | 2.11E-02 | 0.78 |
| PC 35:6 (2) | 4.95E-02 | 1.31E-01 | 3.85 | | LPG 18:1 | 3.25E-03 | 1.35E-02 | 2.10 | | LPC 18:2 (3) | 8.03E-03 | 2.72E-02 | 0.73 |
| PE 36:2;2O (PE 18:2\_18:0;2O) (2) | 3.57E-02 | 1.20E-01 | 2.98 | | CAR 15:0 | 2.33E-03 | 6.34E-03 | 2.07 | | LPC 17:1 | 1.93E-02 | 3.34E-02 | 0.72 |
| LPC 16:0 (2) | 4.69E-02 | 7.39E-02 | 2.56 | | LPE O-18:2 | 1.85E-03 | 7.25E-03 | 2.06 | | LPC 20:4 (2) | 1.52E-02 | 2.68E-02 | 0.71 |
| PC O-32:1 (PC O-16:0\_16:1) | 3.01E-02 | 1.09E-01 | 2.17 | | FA 15:3;2O | 2.23E-02 | 4.50E-02 | 2.03 | | LPC 20:3 | 2.73E-02 | 5.98E-02 | 0.69 |
| DMPE 34:2 (DMPE 16:0\_18:2) | 4.69E-02 | 1.17E-01 | 1.76 | | CAR 18:0 | 9.05E-03 | 2.35E-02 | 1.98 | | LPG 18:1 | 2.05E-02 | 2.75E-02 | 0.67 |
| 4-Imidazoleacrylic acid | 2.84E-02 | 6.80E-02 | 1.69 | | CAR 14:1 | 3.92E-02 | 1.48E-01 | 1.98 | | PC 44:12 | 4.22E-02 | 1.16E-01 | 0.64 |
| PE-Cer 37:9;3O | 4.48E-03 | 1.41E-02 | 1.65 | | FA 20:4;2O | 7.45E-03 | 1.67E-02 | 1.98 | | CAR 18:1 | 5.33E-03 | 1.50E-02 | 0.62 |
| FA 15:3;2O | 4.21E-02 | 7.85E-02 | 1.61 | | CAR 16:1 | 5.52E-03 | 1.51E-02 | 1.96 | | CAR 15:0 | 1.62E-02 | 2.85E-02 | 0.62 |
| PE 36:2;O (PE 18:1\_18:1;O) | 4.69E-02 | 1.31E-01 | 1.60 | | LPC 20:1 (2) | 1.46E-03 | 3.72E-03 | 1.90 | | CAR 18:2 | 4.44E-02 | 9.41E-02 | 0.61 |
| PE 36:2 (PE 18:0\_18:2) | 3.57E-02 | 9.61E-02 | 1.58 | | PG 36:3 (PG 18:1\_18:2) (2) | 2.63E-02 | 4.47E-02 | 1.88 | | CAR 18:0 | 2.05E-02 | 3.46E-02 | 0.59 |
| LPC 14:0 | 1.45E-02 | 3.31E-02 | 1.54 | | LPC 20:0 | 3.92E-02 | 1.02E-01 | 1.86 | | CAR 16:1 | 5.33E-03 | 9.77E-03 | 0.58 |
| LPC 19:0 (2) | 1.25E-03 | 3.92E-03 | 1.50 | | LPC 14:0 | 5.52E-03 | 9.34E-03 | 1.79 | | CAR 17:0 | 1.19E-02 | 2.41E-02 | 0.55 |
| PC O-35:5 (2) | 4.95E-02 | 1.50E-01 | 1.47 | | CAR 18:1 | 5.52E-03 | 1.10E-02 | 1.78 | | FA 20:4;2O | 9.78E-03 | 2.40E-02 | 0.54 |
| LPE O-18:2 | 1.99E-02 | 2.87E-02 | 1.45 | | LPE O-18:1 | 3.92E-02 | 5.42E-02 | 1.77 | | FA 18:1;2O (1) | 5.71E-03 | 2.04E-02 | 0.54 |
| LPC 20:1 (2) | 3.77E-02 | 6.75E-02 | 1.43 | | CAR 18:2 | 2.63E-02 | 6.23E-02 | 1.74 | | PI 38:5|PI 18:1\_20:4 (1) | 4.00E-02 | 1.20E-01 | 0.53 |
| LPC 15:0/0:0 | 4.69E-02 | 8.08E-02 | 1.38 | | LPC 19:0 (1) | 8.22E-03 | 1.06E-02 | 1.72 | | Sphingosine 1-phosphate | 3.23E-02 | 6.26E-02 | 0.51 |
| LPE O-18:1 | 3.57E-02 | 8.58E-02 | 1.32 | | LPC 19:0 (2) | 5.36E-04 | 1.06E-03 | 1.71 | | CAR 16:0 | 2.38E-03 | 5.21E-03 | 0.51 |
| LPE O-16:1 | 3.57E-02 | 8.66E-02 | 1.27 | | LPC 15:0/0:0 | 9.05E-03 | 2.11E-02 | 1.69 | | CAR 14:0 | 6.12E-03 | 1.31E-02 | 0.51 |
| FA 18:0;2O | 3.37E-02 | 7.39E-02 | 1.23 | | LPE O-16:1 | 2.23E-02 | 2.93E-02 | 1.69 | | FA 20:4;3O (2) | 2.73E-02 | 3.86E-02 | 0.47 |
| NAE 7:0 | 1.28E-02 | 3.74E-02 | 1.17 | | FA 16:0;2O | 2.03E-04 | 7.04E-04 | 1.63 | | SM 34:2;3O (1) | 1.34E-02 | 4.09E-02 | 0.47 |
| CL 80:1 (CL 18:0\_18:0\_26:0\_18:1) | 4.21E-02 | 8.86E-02 | 1.08 | | LPC 19:1 | 1.88E-02 | 4.20E-02 | 1.60 | | FA 20:3;3O | 8.58E-03 | 3.17E-02 | 0.46 |
| LNAPE 38:5 (NAPE 20:4/N-18:1) | 1.99E-02 | 5.38E-02 | 0.91 | | LPC 16:0 (3) | 2.61E-03 | 1.01E-02 | 1.57 | | ST 27:1;O;S (1) | 3.41E-02 | 1.28E-01 | 0.41 |
| NAE 12:0 | 1.43E-04 | 5.65E-04 | 0.70 | | OxFA 18:0;(2OH) | 3.10E-02 | 4.23E-02 | 1.55 | | FA 20:3;O | 4.31E-03 | 9.67E-03 | 0.40 |
| PE 38:3 (PE 18:0\_20:3) (2) | 3.57E-02 | 4.81E-02 | 0.34 | | PE-Cer 37:9;3O | 1.32E-02 | 2.13E-02 | 1.55 | | PG 36:3 (PG 18:1\_18:2) (2) | 1.72E-02 | 3.79E-02 | 0.39 |
| | | | | | LPC 17:1 | 2.63E-02 | 6.37E-02 | 1.52 | | FA 22:5;O (1) | 1.49E-03 | 2.70E-03 | 0.39 |
| | | | | | LPC 18:1 | 1.58E-02 | 3.55E-02 | 1.45 | | PE 40:5;O (PE 22:4\_18:1;O) (2) | 4.00E-02 | 9.56E-02 | 0.35 |
| | | | | | LPC 20:2 | 4.23E-02 | 1.23E-01 | 1.44 | | FA 22:5;3O | 2.38E-03 | 1.03E-02 | 0.35 |
| | | | | | LPC 17:0 | 2.63E-02 | 8.98E-02 | 1.42 | | FA 20:3;4O | 1.07E-03 | 3.71E-03 | 0.34 |
| | | | | | FA 19:5;2O (3) | 3.10E-02 | 8.73E-02 | 1.37 | | FA 20:4;3O (1) | 2.57E-03 | 6.75E-03 | 0.34 |
| | | | | | FA 18:0;2O | 1.09E-02 | 1.72E-02 | 1.33 | | PG 40:7 (PG 18:1\_22:6) (2) | 8.58E-03 | 5.16E-02 | 0.34 |
| | | | | | FA 15:4 | 4.92E-02 | 1.41E-01 | 1.30 | | FA 20:4;O (1) | 3.73E-03 | 9.03E-03 | 0.34 |
| | | | | | CAR 6:0 | 2.42E-02 | 1.34E-01 | 1.07 | | FA 22:6;O | 1.74E-03 | 4.53E-03 | 0.31 |
| | | | | | PI 37:4|PI 17:0\_20:4 | 4.56E-02 | 9.87E-02 | 0.91 | | FA 20:5;O | 5.33E-03 | 1.22E-02 | 0.30 |
| | | | | | PE O-38:4 (PE O-18:1\_20:3) (2) | 3.35E-02 | 2.24E-01 | 0.71 | | FA 22:4;O | 6.12E-03 | 1.57E-02 | 0.30 |
| | | | | | NAE 12:0 | 2.56E-05 | 1.11E-04 | 0.63 | | PE O-34:3 (PE O-16:1\_18:2) (1) | 1.37E-03 | 3.08E-03 | 0.28 |
| | | | | | SM 40:2;2O (1) | 4.23E-02 | 1.47E-01 | 0.54 | | FA 20:5;2O (2) | 2.35E-05 | 2.81E-04 | 0.26 |
| | | | | | PE 38:5;O (PE 20:4\_18:1;O) (1) | 1.44E-02 | 4.33E-02 | 0.49 | | FA 22:5;O (2) | 5.39E-04 | 1.47E-03 | 0.21 |
| | | | | | PI 38:3 (PI 18:0\_20:3) (5) | 1.20E-02 | 1.26E-02 | 0.45 | | FA 20:4;O (2) | 5.89E-04 | 7.04E-03 | 0.11 |
| | | | | | PE O-40:6 (PE O-18:1\_22:5) (3) | 7.45E-03 | 1.76E-02 | 0.45 | | | | | |
| | | | | | SM 42:3;2O (SM 18:1;2O/24:2) (1) | 3.62E-03 | 1.41E-02 | 0.45 | | | | | |
| | | | | | PE O-38:4 (PE O-18:1\_20:3) (1) | 1.44E-02 | 1.05E-01 | 0.45 | | | | | |
| | | | | | PE O-38:4 (PE O-18:0\_20:4) | 3.10E-02 | 9.03E-02 | 0.45 | | | | | |
| | | | | | PE 42:7;O (PE 20:4\_22:3;O) | 2.63E-02 | 4.68E-02 | 0.44 | | | | | |
| | | | | | HexCer 34:1;2O | 3.10E-02 | 8.82E-02 | 0.41 | | | | | |
| | | | | | PE O-40:9 (PE O-18:3\_22:6) | 1.44E-02 | 9.34E-02 | 0.29 | | | | | |
| | | | | | PE 38:3 (PE 18:0\_20:3) (3) | 8.22E-03 | 3.36E-02 | 0.25 | | | | | |
| | | | | | SM 36:1;2O | 1.20E-02 | 3.82E-02 | 0.22 | | | | | |
| | | | | | PE 38:3 (PE 18:0\_20:3) (2) | 1.44E-02 | 4.99E-02 | 0.20 | | | | | |
| | | | | | PI 38:4 (PI 18:0\_20:4) (6) | 1.09E-02 | 3.61E-02 | 0.18 | | | | | |

## Slide 7
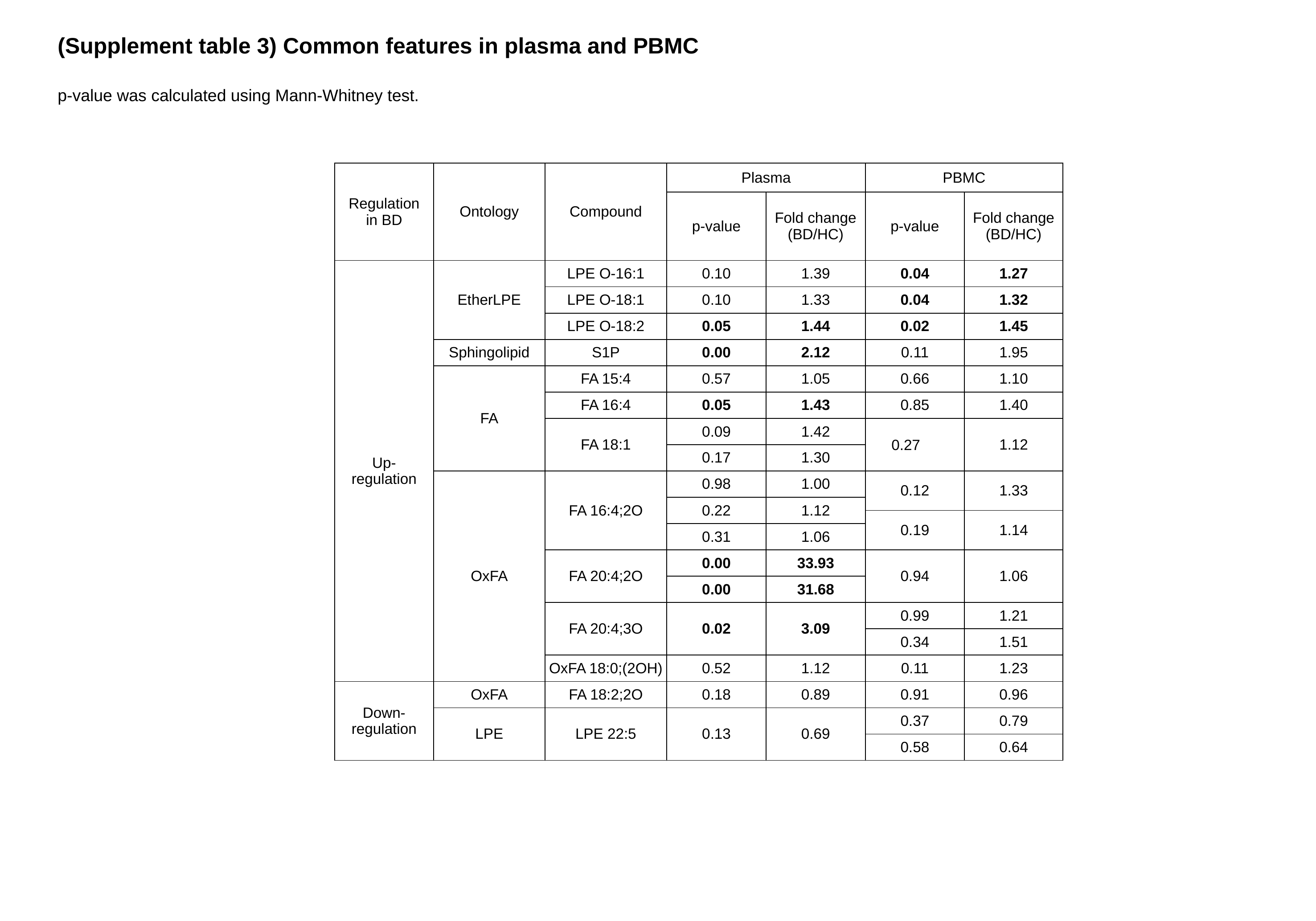

(Supplement table 3) Common features in plasma and PBMC
p-value was calculated using Mann-Whitney test.
| Regulation in BD | Ontology | Compound | Plasma | | PBMC | |
| --- | --- | --- | --- | --- | --- | --- |
| | | | p-value | Fold change (BD/HC) | p-value | Fold change (BD/HC) |
| Up- regulation | EtherLPE | LPE O-16:1 | 0.10 | 1.39 | 0.04 | 1.27 |
| | | LPE O-18:1 | 0.10 | 1.33 | 0.04 | 1.32 |
| | | LPE O-18:2 | 0.05 | 1.44 | 0.02 | 1.45 |
| | Sphingolipid | S1P | 0.00 | 2.12 | 0.11 | 1.95 |
| | FA | FA 15:4 | 0.57 | 1.05 | 0.66 | 1.10 |
| | | FA 16:4 | 0.05 | 1.43 | 0.85 | 1.40 |
| | | FA 18:1 | 0.09 | 1.42 | 0.27 | 1.12 |
| | | | 0.17 | 1.30 | | |
| | OxFA | FA 16:4;2O | 0.98 | 1.00 | 0.12 | 1.33 |
| | | | 0.22 | 1.12 | 0.19 | 1.14 |
| | | | | | 0.19 | 1.14 |
| | | | 0.31 | 1.06 | 0.19 | 1.14 |
| | | FA 20:4;2O | 0.00 | 33.93 | 0.94 | 1.06 |
| | | | 0.00 | 31.68 | | |
| | | FA 20:4;3O | 0.02 | 3.09 | 0.99 | 1.21 |
| | | | | | 0.34 | 1.51 |
| | | OxFA 18:0;(2OH) | 0.52 | 1.12 | 0.11 | 1.23 |
| Down- regulation | OxFA | FA 18:2;2O | 0.18 | 0.89 | 0.91 | 0.96 |
| | LPE | LPE 22:5 | 0.13 | 0.69 | 0.37 | 0.79 |
| | | | | | 0.58 | 0.64 |

## Slide 8
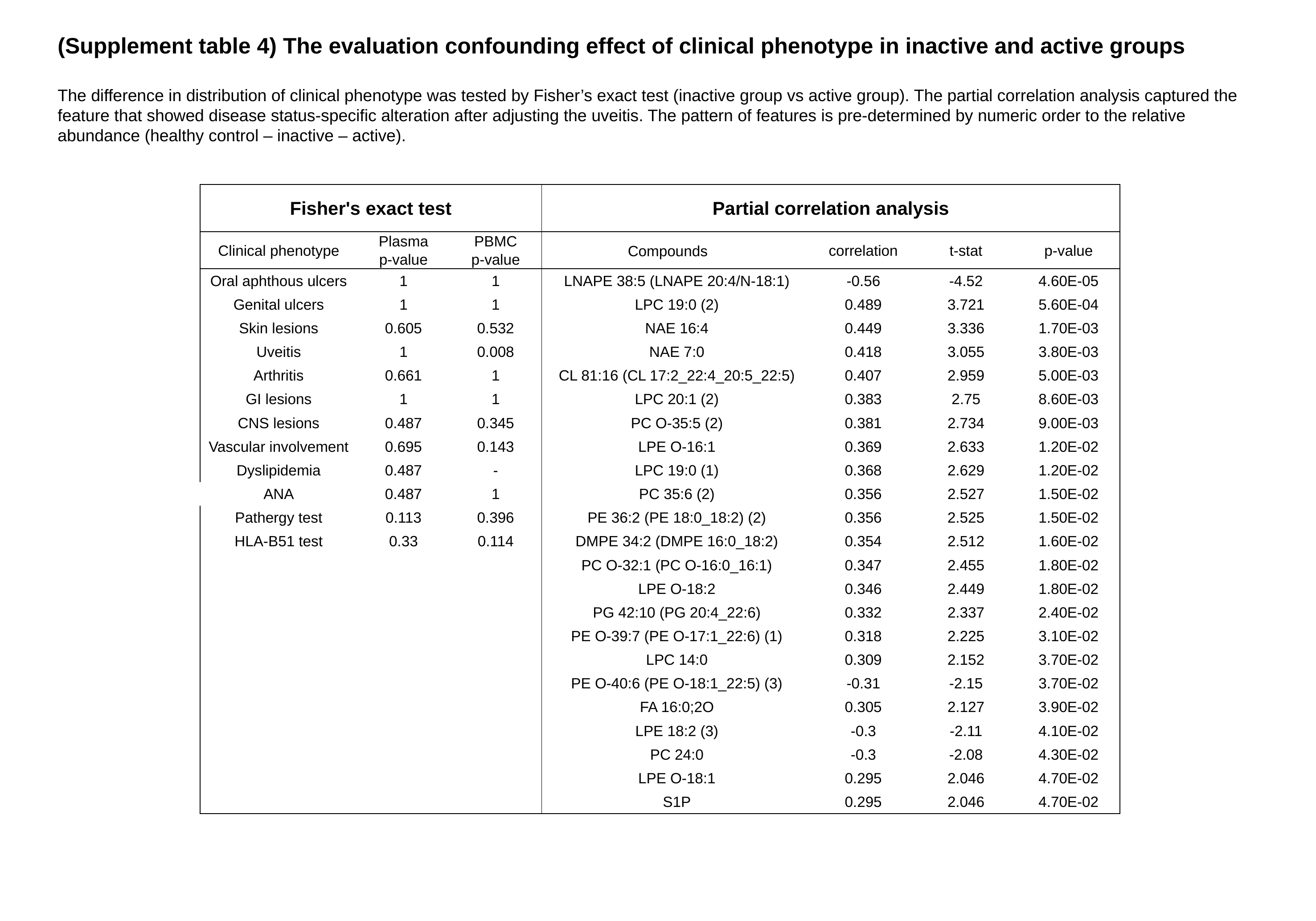

(Supplement table 4) The evaluation confounding effect of clinical phenotype in inactive and active groups
The difference in distribution of clinical phenotype was tested by Fisher’s exact test (inactive group vs active group). The partial correlation analysis captured the feature that showed disease status-specific alteration after adjusting the uveitis. The pattern of features is pre-determined by numeric order to the relative abundance (healthy control – inactive – active).
| Fisher's exact test | | | Partial correlation analysis | | | |
| --- | --- | --- | --- | --- | --- | --- |
| Clinical phenotype | Plasma | PBMC | Compounds | correlation | t-stat | p-value |
| | p-value | p-value | | | | |
| Oral aphthous ulcers | 1 | 1 | LNAPE 38:5 (LNAPE 20:4/N-18:1) | -0.56 | -4.52 | 4.60E-05 |
| Genital ulcers | 1 | 1 | LPC 19:0 (2) | 0.489 | 3.721 | 5.60E-04 |
| Skin lesions | 0.605 | 0.532 | NAE 16:4 | 0.449 | 3.336 | 1.70E-03 |
| Uveitis | 1 | 0.008 | NAE 7:0 | 0.418 | 3.055 | 3.80E-03 |
| Arthritis | 0.661 | 1 | CL 81:16 (CL 17:2\_22:4\_20:5\_22:5) | 0.407 | 2.959 | 5.00E-03 |
| GI lesions | 1 | 1 | LPC 20:1 (2) | 0.383 | 2.75 | 8.60E-03 |
| CNS lesions | 0.487 | 0.345 | PC O-35:5 (2) | 0.381 | 2.734 | 9.00E-03 |
| Vascular involvement | 0.695 | 0.143 | LPE O-16:1 | 0.369 | 2.633 | 1.20E-02 |
| Dyslipidemia | 0.487 | - | LPC 19:0 (1) | 0.368 | 2.629 | 1.20E-02 |
| ANA | 0.487 | 1 | PC 35:6 (2) | 0.356 | 2.527 | 1.50E-02 |
| Pathergy test | 0.113 | 0.396 | PE 36:2 (PE 18:0\_18:2) (2) | 0.356 | 2.525 | 1.50E-02 |
| HLA-B51 test | 0.33 | 0.114 | DMPE 34:2 (DMPE 16:0\_18:2) | 0.354 | 2.512 | 1.60E-02 |
| | | | PC O-32:1 (PC O-16:0\_16:1) | 0.347 | 2.455 | 1.80E-02 |
| | | | LPE O-18:2 | 0.346 | 2.449 | 1.80E-02 |
| | | | PG 42:10 (PG 20:4\_22:6) | 0.332 | 2.337 | 2.40E-02 |
| | | | PE O-39:7 (PE O-17:1\_22:6) (1) | 0.318 | 2.225 | 3.10E-02 |
| | | | LPC 14:0 | 0.309 | 2.152 | 3.70E-02 |
| | | | PE O-40:6 (PE O-18:1\_22:5) (3) | -0.31 | -2.15 | 3.70E-02 |
| | | | FA 16:0;2O | 0.305 | 2.127 | 3.90E-02 |
| | | | LPE 18:2 (3) | -0.3 | -2.11 | 4.10E-02 |
| | | | PC 24:0 | -0.3 | -2.08 | 4.30E-02 |
| | | | LPE O-18:1 | 0.295 | 2.046 | 4.70E-02 |
| | | | S1P | 0.295 | 2.046 | 4.70E-02 |
